# Decomposing DEA self-efficiency: A cross-efficiency-based quantity–price framework

**DOI:** 10.1371/journal.pone.0355649

**Published:** 2026-08-10

**Authors:** Yanjun Liu, Zanfu Ma, Hao Zhan

**Affiliations:** 1 School of Transport and Logistics, Guangzhou Railway Polytechnic, Guangzhou, China; 2 School of Economics and Management, Wenzhou University of Technology, Wenzhou, China; University of Tehran, IRAN, ISLAMIC REPUBLIC OF

## Abstract

Data Envelopment Analysis (DEA) self-efficiency scores are widely used for measuring relative performance. Although it has long been recognized that DEA efficiency depends on endogenous weight systems and the underlying production technology, the mechanisms through which these factors jointly contribute to observed efficiency differences remain insufficiently understood and difficult to quantify. Existing cross-efficiency studies have primarily focused on peer-appraisal aggregation and ranking performance, while the informational structure embedded in cross-efficiency matrices has received comparatively little attention. This study exploits the dual informational organization of cross-efficiency matrices to develop a quantity–price decomposition framework for DEA self-efficiency. Row-wise comparisons preserve common valuation systems and predominantly reflect production-position information, whereas column-wise comparisons preserve common quantity bundles and predominantly reflect endogenous valuation information generated by alternative shadow-price systems. Based on four cross-efficiency elements, a reciprocal Quantity Index (QI) and a reciprocal Price Index (PI) are constructed to decompose pairwise self-efficiency differences into quantity-related and valuation-related components. The proposed decomposition provides an exact algebraic representation of self-efficiency differences, while its interpretation should be understood as a dominant-information decomposition within the DEA framework because every cross-efficiency element is jointly determined by observed quantities and endogenous shadow prices. Theoretical properties of the proposed framework are established, including positivity, reciprocity, spectral equivalence, and the existence and uniqueness of principal eigenvectors. An empirical illustration using data from Chinese cities demonstrates how production-position information and endogenous valuation information can be extracted from cross-efficiency matrices and how alternative technological assumptions simultaneously reshape quantity and valuation structures. Rather than proposing a new cross-efficiency evaluation method, this study provides an interpretive framework for quantifying how endogenous weighting systems and technological assumptions contribute to DEA self-efficiency formation. By shifting attention from peer-appraisal aggregation to the informational structure underlying DEA self-efficiency, the study offers an additional perspective for understanding model dependence, scale efficiency, and the formation mechanism of DEA self-efficiency.

## Introduction

Data Envelopment Analysis (DEA), originally proposed by Charnes et al. [1], has become one of the most widely used nonparametric methods for evaluating the relative performance of decision-making units (DMUs) operating with multiple inputs and outputs. Owing to its ability to endogenously determine input and output weights without imposing a priori price information or functional assumptions, DEA has been extensively applied in banking, healthcare, education, transportation, energy, and public-sector performance evaluation.

A distinctive feature of DEA is that efficiency scores are generated through endogenous shadow-price systems. Consequently, DEA efficiency is fundamentally different from conventional productivity indicators based on externally specified aggregation rules. The estimated efficiency of a DMU depends not only on its observed input-output bundle but also on the production technology used to construct the production possibility set and the endogenous valuation system implied by the optimization model. Alterations in returns-to-scale assumptions, orientation specifications, weight restrictions, or secondary-goal formulations may substantially change efficiency estimates even when the underlying production data remain unchanged. DEA efficiency should therefore be viewed as both technology-dependent and weight-dependent.

Although this dependence has long been recognized in the DEA literature, relatively little is known about how technology and endogenous valuation systems jointly contribute to observed self-efficiency differences and, more importantly, how their effects can be quantified. Existing studies generally regard model dependence and weight flexibility as sources of sensitivity, robustness concerns, or ranking instability. Consequently, DEA self-efficiency is usually interpreted as a single composite measure, whereas the informational mechanisms responsible for its formation remain largely hidden.

A substantial literature has developed decomposition frameworks to improve the interpretability of DEA-based performance measures. Existing studies have decomposed scale effects [2], productivity changes [3], network structures [4], and variable contributions [5], thereby considerably enriching the explanatory capability of DEA. Nevertheless, most decomposition approaches operate directly on efficiency scores, productivity indices, or model components and rarely investigate the informational structure embedded in cross-efficiency systems. A detailed review of this literature is provided in Section 2.

Cross-efficiency evaluation, originally introduced by Sexton et al. [6], extends conventional DEA by allowing each DMU to be evaluated not only by its own optimal weights but also by the optimal weights of its peers. The resulting cross-efficiency matrix contains considerably richer information than a vector of self-efficiency scores because each matrix element simultaneously embodies two sources of information: the relative production position of the evaluated DMU and the endogenous valuation system represented by DEA shadow prices. Existing cross-efficiency studies have primarily focused on peer-appraisal aggregation, ranking discrimination, secondary-goal formulations, consensus evaluation, and weight restrictions. Comparatively little attention has been devoted to the informational organization of the cross-efficiency matrix itself and to whether the formation of self-efficiency differences can be further understood through complementary production-position and valuation dimensions.

Based on this perspective, this study develops a quantity–price decomposition framework for DEA self-efficiency based on cross-efficiency matrices. Reciprocal quantity and price comparison matrices are constructed by exploiting the row-wise and column-wise informational organization of cross-efficiency evaluations. The resulting Quantity Index (QI) predominantly summarizes production-position information under common valuation systems, whereas the Price Index (PI) predominantly summarizes endogenous valuation information under common quantity bundles. Principal eigenvectors of the reciprocal comparison matrices are then extracted to construct globally comparable quantity and price measures.

The proposed decomposition should be interpreted carefully. At the algebraic level, pairwise self-efficiency differences can be represented exactly through quantity and price ratios. However, because every cross-efficiency element is jointly determined by observed quantities and endogenous shadow prices, neither index constitutes a completely purified measure of quantity or valuation information. Instead, the proposed framework provides a dominant-information decomposition that emphasizes production-position effects and endogenous valuation effects while preserving their intrinsic interdependence.

Despite this expanded informational basis, existing studies have primarily regarded the cross-efficiency matrix as an intermediate tool for ranking DMUs, aggregating peer evaluations, or improving discrimination among efficiency scores. Consequently, the matrix structure itself, including the reciprocal relationships among cross-evaluations generated by heterogeneous DEA weight systems, has received relatively limited attention. From this perspective, cross-efficiency matrices contain richer information than a final ranking index: they represent a relational system of endogenous valuation structures among DMUs, where differences in self-efficiency outcomes may arise from both relative production positions and valuation structures induced by DEA weights.

Accordingly, this study does not attempt to construct a new DEA optimization model or replace existing cross-efficiency evaluation procedures. Instead, it investigates whether the information embedded in cross-efficiency matrices can be further organized and decomposed into interpretable dimensions. Specifically, the quantity–price decomposition framework identifies two complementary dimensions underlying DEA self-efficiency formation: a quantity-related dimension associated with relative production positions and a price-related dimension associated with endogenous valuation structures generated by DEA weight systems.

This perspective is consistent with the broader tradition of decomposition analysis in DEA and related economic measurement. Similar to traditional DEA decomposition approaches, such as the decomposition of technical efficiency into pure technical efficiency and scale efficiency, the proposed framework seeks to uncover different sources contributing to observed efficiency differences. Therefore, the contribution of this study does not lie in proposing another DEA efficiency measure, but in extending the analytical use of cross-efficiency matrices from ranking-oriented representations toward structural interpretations of how DEA self-efficiency is formed.

The contributions of this study are threefold. First, it proposes a quantity–price decomposition framework that reveals two complementary dimensions embedded in DEA self-efficiency formation. Second, it establishes a structural connection between DEA cross-efficiency matrices and AHP-type reciprocal comparison structures, providing a new perspective for understanding cross-efficiency information beyond ranking. Third, it demonstrates how the proposed framework can be used to analyze technology-induced differences in DEA self-efficiency formation and the associated quantity and valuation effects.

The remainder of the paper is organized as follows. Section 2 reviews the related literature and identifies the existing research gap. Section 3 develops the quantity-price decomposition framework and establishes its theoretical properties. Section 4 presents an empirical illustration and investigates the quantity and valuation structures extracted from cross-efficiency matrices under alternative technologies. Section 5 discusses the implications of the proposed framework for understanding DEA self-efficiency formation and scale efficiency. Section 6 concludes.

## Literature review

### DEA efficiency decomposition literature

Since the introduction of Data Envelopment Analysis (DEA) by Charnes et al. [1], efficiency measurement has become one of the most influential research streams in operations research, management science, and performance evaluation. While early DEA studies primarily focused on identifying efficient frontiers and measuring relative efficiency, subsequent research increasingly emphasized understanding the underlying sources of efficiency differences. Consequently, a substantial body of literature has developed various decomposition frameworks to enhance the interpretability of DEA results.

One important stream of research concerns productivity and efficiency decomposition. The earliest decomposition idea in DEA can be traced to the BCC model of Banker et al. [2], which demonstrated that overall technical efficiency under constant returns to scale can be decomposed into pure technical efficiency and scale efficiency. Extending decomposition analysis from a static to a dynamic setting, Färe et al. [3] proposed the Malmquist Productivity Index (MPI), which decomposes total factor productivity change into efficiency change and technological change. This framework established the foundation for analyzing productivity dynamics and has subsequently been extended to accommodate undesirable outputs, environmental factors, and global technologies. Representative developments include the Malmquist-Luenberger productivity index [7], the global Malmquist productivity index [8], and the global Malmquist-Luenberger index [9]. Numerous empirical studies have employed these decomposition paradigms to investigate productivity growth and technological progress across industries and countries. Collectively, this stream of literature demonstrates that aggregate productivity changes can be interpreted through the decomposition of underlying technological and efficiency components.

A second stream focuses on structural and process decomposition within complex production systems. Recognizing that many real-world organizations operate through interconnected stages and temporal linkages, researchers developed network DEA models capable of decomposing overall efficiency into stage-specific components. The relational network DEA model proposed by Färe and Grosskopf [4], the decomposition framework developed by Kao [10], and the network SBM model introduced by Tone and Tsutsui [11] represent important milestones in this line of research. Extending the SBM framework, Kao [12] proposed a general network SBM model and demonstrated that system efficiency can be decomposed into a weighted average of process efficiencies for general network structures. Subsequently, Kao [13] further distinguished between efficiency decomposition and efficiency aggregation and established a theoretical framework for characterizing the relationships between system efficiency and subsystem efficiencies in network DEA. These studies substantially improved the interpretability of DEA by revealing how internal production structures contribute to aggregate efficiency performance.

A third stream concerns attribution and contribution decomposition. The development of non-radial DEA models, particularly the Slack-Based Measure (SBM) proposed by Tone [14], substantially enriched decomposition analysis by enabling inefficiency to be directly attributed to input excesses and output shortfalls. Subsequent studies further decomposed inefficiency into input-specific and output-specific components [15], thereby providing more detailed diagnostic information regarding the sources of performance deficiencies. More recently, decomposition methods based on cooperative game theory have attracted considerable attention. Drawing on the Shapley value framework, researchers have developed methods to quantify the contributions of individual variables, environmental factors, and intermediate products to DEA efficiency [5,16]. These approaches systematically evaluate marginal contributions while accounting for interaction effects among variables and demonstrate that DEA outcomes can be interpreted through the decomposition of underlying informational components rather than through aggregate efficiency measures alone.

Another emerging stream concerns informational representation and variable-selection studies in DEA. Since DEA efficiency estimates are highly sensitive to the specification of inputs and outputs, considerable attention has been devoted to identifying influential variables and alleviating the curse of dimensionality. Existing approaches include mathematical optimization-based feature selection and cardinality-constrained DEA models [17,18], as well as recent integrations of machine-learning techniques such as LASSO regularization and high-dimensional sparse modeling with DEA frameworks [19,20]. These studies suggest that efficiency estimates depend not only on observed production data and technological assumptions but also on how production information is represented, selected, and organized within the evaluation framework. The informational representation perspective therefore provides an important conceptual foundation for investigating whether cross-efficiency matrices themselves contain interpretable structural information.

Despite these important advances, existing DEA decomposition studies predominantly focus on productivity change, network structures, slack distributions, variable contributions, or informational representations of production activities. The decomposition is generally performed directly on productivity indices, efficiency scores, distance functions, slacks, or model components after the evaluation process has been completed. Consequently, existing studies provide valuable explanations of observed efficiency outcomes but offer limited insights into the informational mechanisms through which DEA self-efficiency itself is generated.

In particular, comparatively little attention has been devoted to the informational organization embedded in cross-efficiency systems. Existing decomposition frameworks rarely examine whether cross-efficiency matrices preserve interpretable structural information regarding the formation of self-efficiency and whether such information can be organized into complementary production-position and endogenous valuation dimensions. Since DEA self-efficiency is jointly shaped by production technologies, relative production positions, and endogenous shadow-price systems, understanding how these informational components interact represents an important but largely unexplored research question.

This limitation motivates the present study. Rather than decomposing efficiency outcomes directly, the proposed framework exploits the informational structure embedded in cross-efficiency matrices to construct quantity and price indices that quantify production-position effects and endogenous valuation effects underlying DEA self-efficiency formation.

### Cross-efficiency evaluation literature

#### Classical cross-efficiency evaluation.

Cross-efficiency evaluation, originally introduced by Sexton et al. [6], represents one of the most influential developments for addressing the weight variability inherent in conventional DEA models. By allowing each decision-making unit (DMU) to evaluate not only itself but also all peer DMUs using its optimal weight system, cross-efficiency combines self-evaluation and peer-evaluation information and often improves ranking discrimination.

Doyle and Green [21] recognized that multiple optimal DEA solutions may generate multiple cross-efficiency matrices and consequently proposed aggressive and benevolent secondary-goal formulations. Their work established the theoretical foundation of modern cross-efficiency evaluation.

Subsequent research developed numerous extensions designed to improve weight selection, ranking stability, and aggregation performance. Representative contributions include secondary-goal programming, entropy-based weighting, voting-based aggregation methods, and various consensus procedures [22–36].

Despite these advances, two fundamental issues remain unresolved. First, the existence of multiple optimal DEA weights inevitably leads to non-unique cross-efficiency matrices. Second, aggregation rules themselves introduce additional value judgments, and alternative aggregation schemes may generate conflicting rankings and inconsistent performance assessments [37].

Cross-efficiency evaluation under variable returns to scale (VRS) has also attracted considerable attention. Lim and Zhu [38] demonstrated that conventional input-oriented VRS cross-efficiency models may generate negative cross-efficiency values and proposed corrective procedures to address this problem.

#### Fairness, consensus, and behavioral extensions.

Recent cross-efficiency research has shifted from purely ranking-oriented objectives toward fairness, consensus formation, and behavioral considerations. Pan et al. [39] introduced bounded rationality into cross-efficiency aggregation procedures, providing a behavioral interpretation of peer-evaluation processes. Zhang et al. [40] incorporated fairness mentality and group-consensus mechanisms into cross-efficiency aggregation, while Hao et al. [41] further developed interval cross-efficiency models that integrate consensus acceptability analysis within group decision-making environments.

These developments indicate that modern cross-efficiency evaluation increasingly emphasizes behavioral realism and collective acceptability rather than simple ranking discrimination.

#### Explainable and decision-oriented cross-efficiency models.

As efficiency evaluation becomes increasingly important for managerial decision making, researchers have begun to emphasize interpretability and explainability. Yang et al. [42] proposed a centralized cross-efficiency framework enhanced by explainable artificial intelligence (XAI), while Ma and Yin [43] incorporated expert preferences into cross-efficiency evaluation.

These studies suggest that efficiency analysis should not only identify performance differences but also explain their underlying causes. Nevertheless, existing explainability studies remain primarily focused on ranking interpretation rather than on the informational structure embedded within cross-efficiency matrices themselves.

#### Network and hybrid cross-efficiency models.

A further development concerns the extension of cross-efficiency evaluation to network and multi-stage production systems. Ganji et al. [44], Chen et al. [45], and Liu et al. [46] extended cross-efficiency concepts to double-frontier, network, and two-stage DEA structures. Simultaneously, hybrid approaches combining cross-efficiency with BWM, evidential reasoning, entropy weighting, portfolio optimization, and game-theoretic methods have become increasingly common [47–49].

Collectively, these studies demonstrate that cross-efficiency evaluation has evolved from a ranking methodology into a versatile analytical framework capable of addressing increasingly complex decision environments.

#### Summary of cross-efficiency research.

The evolution of cross-efficiency evaluation reveals a clear transition from ranking-oriented analysis toward fairness, consensus formation, explainable decision making, behavioral realism, sustainability assessment, and complex-system evaluation.

Nevertheless, despite these methodological advances, most studies continue to treat the cross-efficiency matrix primarily as a computational device for generating rankings, consensus scores, or decision recommendations. The rich information embedded in the matrix is ultimately compressed into a single performance statistic.

More importantly, existing studies mainly focus on improving ranking performance rather than explaining how endogenous weight structures influence aggregated outcomes. Consequently, robustness assessments and ranking comparisons are frequently conducted ex post and often lack a unified theoretical foundation for explaining why alternative DEA models generate different efficiency outcomes.

Since each element of a cross-efficiency matrix simultaneously contains production information and valuation information implied by endogenous DEA weights, the matrix may be viewed as an information system containing substantially richer structural information than is conveyed by ranking statistics alone. However, little research has attempted to exploit this information system to explain how peer evaluations collectively generate DEA self-efficiency or how weight structures and technological assumptions jointly shape efficiency formation.

### Valuation structures and weight flexibility in DEA

One of the most distinctive features of DEA is its endogenous weight determination mechanism. Unlike traditional productivity indices that rely on externally specified prices or weights, DEA allows each DMU to select the most favorable weighting scheme. This flexibility enhances discrimination and avoids subjective weight assignment, but it simultaneously generates substantial heterogeneity in valuation structures across DMUs.

The implications of weight flexibility have attracted considerable attention in the DEA literature. Common-weight models [50,51], assurance-region approaches [52], and weight-restriction techniques [53–55] were developed to improve comparability and reduce excessive weight dispersion. Researchers have also investigated the sensitivity of efficiency scores to alternative weighting schemes and data perturbations.

Early studies by Zhu [56] and Seiford and Zhu [57] demonstrated that DEA efficiency classifications may be sensitive to relatively small perturbations of data and model specifications. Kuntz and Scholtes [58] further emphasized the importance of stability considerations in empirical efficiency analysis. Subsequent contributions extended these investigations to interval data, probabilistic settings, and uncertainty-aware DEA frameworks [59–61].

More recently, comprehensive reviews by Peykani et al. [62] and Mergoni et al. [63] highlighted the growing interest in understanding how alternative weighting structures, uncertainty specifications, and modeling assumptions influence DEA outcomes.

Although these studies have substantially improved our understanding of weight restrictions, sensitivity, and robustness in DEA, they primarily examine the reliability and comparability of efficiency scores rather than the informational content embedded in heterogeneous valuation systems themselves.

Consequently, an important question remains unanswered: how can the valuation information generated by endogenous DEA weight systems be systematically extracted, quantified, and interpreted? Addressing this question is essential for understanding why alternative DEA models often generate different efficiency outcomes despite being applied to the same production data.

The present study addresses this issue by utilizing cross-efficiency information to characterize valuation structures implied by endogenous DEA weights and by investigating how valuation effects interact with production-position effects under alternative technological assumptions.

### Scale efficiency and technology effects

Scale efficiency is one of the most fundamental concepts in DEA. Following Banker et al. [2], scale efficiency is commonly defined as


$$\begin{array}{c} SE_{i}=\frac{{TE}_{i}^{CRS}}{{TE}_{i}^{VRS}} \end{array}$$

where ${TE}_{i}^{CRS}$ and ${TE}_{i}^{VRS}$ denote technical efficiency under constant and variable returns-to-scale technologies, respectively.

Under this framework, differences between CRS and VRS efficiency are generally interpreted as the consequence of operating at a non-optimal scale. Although this interpretation has become standard practice in DEA applications, the underlying mechanism deserves further scrutiny.

Transitioning from CRS to VRS simultaneously changes the production possibility set, modifies peer relationships, and alters the feasible valuation systems generated by DEA models. Consequently, observed differences between CRS and VRS efficiency may reflect not only scale effects but also technology-induced repositioning and technology-induced revaluation effects.

Therefore, scale efficiency should be interpreted as a technology-conditioned outcome jointly determined by changes in production positions and endogenous valuation systems. Understanding these mechanisms may contribute to a more informative interpretation of DEA efficiency measures and their intrinsic limitations.

### Research gap and contributions

The foregoing review reveals four important gaps in the existing literature. First, although DEA studies have developed numerous decomposition frameworks for productivity analysis, network structures, slack attribution, and variable contributions, comparatively little attention has been devoted to exploiting cross-efficiency information to decompose and interpret differences in DEA self-efficiency itself.

Second, despite the rapid development of cross-efficiency evaluation, the cross-efficiency matrix has been employed primarily as a computational device for ranking and consensus building. Its informational structure has rarely been investigated as a means of quantifying how production technologies and endogenous weighting systems contribute to observed differences in DEA self-efficiency.

Third, although weight flexibility and valuation diversity are well-recognized characteristics of DEA, existing studies have rarely attempted to provide a quantitative representation of the valuation information generated by endogenous DEA weight systems or to measure the extent to which weight variability contributes to efficiency differences.

Fourth, conventional scale-efficiency measures do not distinguish between technology-induced production repositioning and technology-induced endogenous revaluation. Consequently, the relative contributions of production-position changes and valuation changes induced by alternative technological assumptions remain difficult to disentangle.

To address these gaps, this study develops a technology-conditioned quantity–price decomposition framework based on the informational structure embedded in cross-efficiency matrices.

First, the study treats the cross-efficiency matrix as an information system rather than merely a ranking device and develops a systematic procedure for constructing reciprocal Quantity and Price Indices from row-wise and column-wise cross-efficiency comparisons.

Second, the proposed framework provides quantitative representations of technology-conditioned production-position information and endogenous revaluation information. The resulting decomposition should not be interpreted as a complete separation between quantity effects and valuation effects. Instead, it constitutes a dominant-effect decomposition in which the Quantity Index is predominantly associated with production-position information and the Price Index is predominantly associated with endogenous valuation information.

Third, the framework makes the effects of weight flexibility and technological assumptions measurable and provides an additional perspective for interpreting model dependence and scale efficiency in DEA. Rather than rediscovering that DEA efficiency depends on endogenous weights and production technologies, the proposed approach quantifies how alternative technological assumptions simultaneously alter production-position information and valuation structures and thereby contribute to observed differences in DEA self-efficiency.

## Constructing the quantity index of DEA efficiency

This section develops the proposed quantity–price decomposition framework by reconsidering DEA self-efficiency from the perspective of index number theory within the general class of ratio-form radial efficiency measures. The cross-efficiency matrix is viewed as an information carrier that contains substantially richer structural information than conventional aggregated peer-appraisal scores. By exploiting the dual informational organization embedded in this matrix, DEA self-efficiency can be decomposed into two distinct components: a quantity index that captures differences in input–output quantities under a common valuation system and a price index that captures revaluation effects generated by alternative endogenous shadow-price systems.

The proposed framework is developed based on conventional DEA self-efficiency evaluation and cross-efficiency construction, and is formally linked to an index-theoretic decomposition of self-efficiency differences. Within this framework, row elements of the cross-efficiency matrix can be interpreted as preserving valuation systems and therefore embedding quantity-related information, whereas column elements preserve quantity bundles and thus reflect valuation-related information. Consequently, the quantity index and the price index provide complementary perspectives for interpreting how DEA self-efficiency is formed and how differences in self-efficiency arise from production quantities and endogenous valuation schemes.

### DEA self-efficiency and cross-efficiency information

Consider a set of *n* DMUs, Let


$$\begin{array}{c} \begin{array}{c} E={\left(e_{ij}\right)}_{n\times n} \end{array} \end{array}$$ (1)

denote the cross-efficiency matrix obtained from a DEA cross-efficiency evaluation model, where $e_{ij}$ represents the efficiency score assigned to DMU-*j* using the optimal weight system of DMU-*i*. The diagonal elements $e_{ii}$ correspond to conventional DEA self-efficiency scores, whereas the off-diagonal elements $e_{ij},i\neq j$ represent peer-evaluation scores.

According to the definition of cross-efficiency given in Equation (1), the column vectors of the cross-efficiency matrix *E* represent all cross-evaluation results of a given DMU under different evaluation standards, whereas the row vectors represent all cross-evaluation results conducted by a given DMU. Specifically, $e_{ji}$ and $e_{jj}$ denote the cross-efficiency scores obtained when DMU-*j* evaluates DMU-*i* and itself, respectively; the difference between them arises from discrepancies in the input and output values of the two DMUs. By contrast, $e_{ii}$ and $e_{ij}$ correspond to the cross-efficiency scores of DMU-*i* evaluated by itself and by DMU-*j*, respectively, and their difference stems from the use of different evaluation standards, or equivalently, from different weight systems supporting the efficiency assessment.

Most existing cross-efficiency studies focus on aggregating peer evaluations to improve ranking discrimination. In contrast, this study treats the cross-efficiency matrix as an information system containing both production-position information and valuation information. Rather than proposing a new cross-efficiency ranking approach, the objective is to develop a decomposition framework that reveals the structural composition of DEA self-efficiency.

It should be emphasized that the proposed framework does not modify the underlying DEA model and does not eliminate the multiplicity of optimal weight systems. Instead, it extracts interpretable structural information from a given cross-efficiency matrix.

### Quantity–price decomposition of self-efficiency formation

The decomposition logic adopted in this study is rooted in the fundamental idea of index number theory, which seeks to decompose changes in aggregate economic measures into interpretable quantity-related and price-related components [64–66]. In classical index number approaches, changes in economic aggregates, such as expenditure, output, and productivity measures, are commonly interpreted as the combined outcome of changes in quantities and prices. This decomposition perspective provides a theoretical basis for distinguishing quantity-related effects from valuation-related effects in measurement and productivity analysis.

The present study extends this decomposition logic to DEA self-efficiency analysis. Unlike conventional economic index numbers, where quantities and prices are externally observed, DEA generates endogenous valuation systems through optimal multiplier selection. Therefore, the quantity-related and price-related components defined here should not be interpreted as physical quantities and market prices. Rather, they represent two structural dimensions embedded in DEA cross-efficiency relationships: one related to relative production positions under a common valuation perspective, and the other related to relative valuation structures induced by alternative DEA weight systems.

Accordingly, the proposed decomposition follows the conceptual spirit of index number theory while adapting it to the endogenous evaluation environment of DEA.

For any pair of DMUs-*i* and *j*, define the self-efficiency ratio as


$$\begin{array}{c} \begin{array}{c} r_{ij}=\frac{e_{ii}}{e_{jj}} \end{array} \end{array}$$ (2)

The ratio measures the relative efficiency difference between two DMUs.

Drawing on the decomposition logic of index number theory, the efficiency ratio can be decomposed into a quantity component and a price component:


$$\begin{array}{c} \begin{array}{c} r_{ij}=q_{ij}p_{ij} \end{array} \end{array}$$ (3)

where $q_{ij}$ denotes the quantity effect and $p_{ij}$ denotes the price effect.

For any pair of DMUs, the construction of the quantity and price components is not arbitrary but follows directly from the informational structure of the cross-efficiency matrix.

Recall that each row of the cross-efficiency matrix is generated by a single evaluator using its own optimal DEA weights. Therefore, all elements in row are supported by an identical valuation system (i.e., the same shadow prices). Variations among these elements mainly reflect differences in the input–output structures of the evaluated DMUs. Consequently, row-wise comparisons naturally isolate quantity-related information.

By contrast, each column of the cross-efficiency matrix corresponds to repeated evaluations of the same DMU under different evaluators’ optimal weight systems. Since the input–output quantities of the evaluated DMU remain fixed, variations among column elements originate exclusively from differences in endogenous valuation systems represented by alternative DEA weights. Therefore, column-wise comparisons mainly reflect valuation-related information.

Accordingly, the quantity index and price index are not introduced as purely algebraic constructions. Instead, they are derived from the dual informational structure embedded in cross-efficiency matrices: row comparisons capture quantity-related effects under a common valuation standard, whereas column comparisons reveal valuation effects under a common quantity bundle.

Using the four cross-efficiency elements $\left(e_{ii},e_{ij},e_{ji},e_{jj}\right)$, the quantity component is defined as


$$\begin{array}{c} \begin{array}{c} q_{ij}={\left(\frac{e_{ji}}{e_{jj}}\frac{e_{ii}}{e_{ij}}\right)}^{\frac{\text{1}}{\text{2}}} \end{array} \end{array}$$ (4)

and the price component is defined as


$$\begin{array}{c} \begin{array}{c} p_{ij}={\left(\frac{e_{ii}}{e_{ji}}\frac{e_{ij}}{e_{jj}}\right)}^{\frac{\text{1}}{\text{2}}} \end{array} \end{array}$$ (5)

**Remark 1.** The proposed quantity–price decomposition is matrix-induced rather than researcher-imposed. Because row elements preserve a common DEA valuation system while column elements correspond to evaluations of the same DMU under different DEA valuation systems, the cross-efficiency matrix naturally contains two complementary types of relational information. Accordingly, the price index should not be interpreted as a market price measure; rather, it captures relative valuation changes generated by alternative DEA multiplier systems. Similarly, the quantity index reflects differences in relative production positions under a common valuation perspective. Therefore, the two indices represent complementary informational dimensions embedded in cross-efficiency relationships rather than completely independent economic quantities.

The terminology of “quantity” and “price” requires further clarification. In conventional index number theory, quantity and price refer to physical quantities and market prices, respectively. However, these concepts have different meanings in the DEA context. The quantity-related component defined in this study does not represent physical quantities in the conventional economic sense; rather, it captures the relative production-position information contained in observed input-output combinations within the DEA evaluation structure. Similarly, the price-related component does not represent market prices. Instead, it reflects the relative valuation intensity generated by endogenous DEA multipliers.

Therefore, the terms quantity and price should be interpreted as structural analogues of index number theory rather than direct economic measurements. The former emphasizes the relative position of DMUs in the production space, whereas the latter emphasizes how the same input-output bundles are valued under different DEA weight systems.

Multiplying Equations (4) and (5) yields


$$\begin{array}{cc} q_{ij}p_{ij}= & {\left(\frac{e_{ji}}{e_{jj}}\frac{e_{ii}}{e_{ij}}\right)}^{\frac{\text{1}}{\text{2}}}{\left(\frac{e_{ii}}{e_{ji}}\frac{e_{ij}}{e_{jj}}\right)}^{\frac{\text{1}}{\text{2}}}\\ = & \;\;\frac{e_{ii}}{e_{jj}}\\ = & \;\;r_{ij} \end{array}$$

Therefore, $r_{ij}=q_{ij}p_{ij}$, and Equation (3) demonstrates that the self-efficiency ratio between any two DMUs can be exactly factorized into a quantity-related component and a price-related component. The quantity-related component reflects differences in relative production positions, whereas the price-related component captures differences arising from endogenous valuation structures embedded in the cross-efficiency system. Consequently, DEA self-efficiency can be interpreted as the joint outcome of production-position information and valuation information.

### Quantity matrix and price matrix

Based on the pairwise decomposition defined above, construct the quantity matrix


$$\begin{array}{c} \begin{array}{c} Q={\left(q_{ij}\right)}_{n\times n} \end{array} \end{array}$$ (6)

and the price matrix


$$\begin{array}{c} \begin{array}{c} P={\left(p_{ij}\right)}_{n\times n} \end{array} \end{array}$$ (7)

These matrices summarize the quantity and price relationships among all DMUs. The following propositions establish their fundamental mathematical properties.

**Proposition 1.** *The quantity matrix Q is a positive reciprocal matrix.*

**Proof.** Since all cross-efficiency scores are strictly positive,


$$\begin{array}{c} q_{ij}>\text{0} \end{array}$$

Furthermore,


$$\begin{array}{c} \begin{array}{ccc} q_{ij} & = & {\left(\frac{e_{ji}}{e_{jj}}\frac{e_{ii}}{e_{ij}}\right)}^{\frac{\text{1}}{\text{2}}}\\ & = & {\left(\frac{e_{ij}}{e_{ii}}\frac{e_{jj}}{e_{ji}}\right)}^{-\frac{\text{1}}{\text{2}}}\\ & = & \;\;\;\;\;\;\;\;\;\;\;\;\;\;\;\;\;\;\;\;\;\frac{\text{1}}{q_{ji}} \end{array} \end{array}$$

Therefore,


$$\begin{array}{c} q_{ij}q_{ji}=\text{1} \end{array}$$

Hence, *Q* is a positive reciprocal matrix.

Because the quantity matrix satisfies positivity and reciprocity, it admits an interpretation analogous to reciprocal judgment matrices in the AHP literature [67]. The reciprocal quantity matrix can therefore be interpreted as an AHP-type judgment matrix. Each column represents a set of relative quantity assessments under one endogenous evaluation criterion generated by an optimal DEA weight system. Consequently, the extraction of the principal eigenvector is matrix-induced rather than researcher-imposed and follows naturally from the established aggregation principle for reciprocal judgment matrices.

**Proposition 2.** *The price matrix P is a positive reciprocal matrix.*

**Proof.** Similarly,


$$\begin{array}{c} p_{ij}>\text{0} \end{array}$$

Moreover,


$$\begin{array}{c} \begin{array}{ccc} p_{ij} & = & {\left(\frac{e_{ii}}{e_{ji}}\frac{e_{ij}}{e_{jj}}\right)}^{\frac{\text{1}}{\text{2}}}\\ & = & {\left(\frac{e_{jj}}{e_{ij}}\frac{e_{ji}}{e_{ii}}\right)}^{-\frac{\text{1}}{\text{2}}}\mathrm{\backslash vspace}1mm\\ & = & \;\;\;\;\;\;\;\;\;\;\;\;\;\;\;\;\;\;\;\;\;\;\frac{\text{1}}{p_{ji}} \end{array} \end{array}$$

Thus,


$$\begin{array}{c} p_{ij}p_{ji}=\text{1} \end{array}$$

Hence, *P* is a positive reciprocal matrix.

The reciprocal structure of *Q* and *P* implies that both matrices contain internally consistent pairwise comparison information. Consequently, global quantity and price measures can be extracted through eigenvector aggregation.

**Proposition 3.** *The quantity matrix* $Q=\left(q_{ij}\right)$ *and the price matrix* $P=\left(p_{ij}\right)$ *possess identical eigenvalues.*

**Proof.** According to Equations (4) and (5), for any pair of DMUs *i* and *j*,


$$\begin{array}{c} q_{ij}=\frac{e_{ii}}{e_{jj}}\frac{\text{1}}{p_{ij}} \end{array}$$

Define the diagonal matrix


$$\begin{array}{c} D=\text{diag}\left(e_{\text{1}\text{1}},e_{\text{2}\text{2}},\cdots ,e_{nn}\right) \end{array}$$

Then,


$$\begin{array}{c} \begin{array}{c} Q=DP^{\prime }D^{-\text{1}} \end{array} \end{array}$$ (8)

That means matrices *Q* and $P^{\prime }$ are similar. Since a matrix and its transpose possess identical spectra,


$$\begin{array}{c} \begin{array}{c} \sigma (Q)=\sigma \left(P^{\prime }\right)=\sigma (P) \end{array} \end{array}$$ (9)

where σ(·) denotes the spectrum of a matrix. Hence, the quantity matrix and the price matrix have exactly the same eigenvalues.

Proposition 3 establishes a spectral equivalence between the quantity matrix and the price matrix. Although the two matrices represent different informational dimensions of DEA self-efficiency, namely production-position effects and valuation-system effects, they possess identical eigenvalues because they are diagonally similar through a transpose transformation. This common spectral structure suggests that both matrices originate from the same cross-efficiency information system. Nevertheless, their distinct principal eigenvectors generate different Quantity and Price Indices, implying that quantity and valuation effects should be viewed as complementary informational representations of DEA self-efficiency formation.

### Construction of quantity and price indices

For each evaluator *j*, the *j*-th column of the quantity matrix,


$$\begin{array}{c} {\left(q_{\text{1}j},q_{\text{2}j}\cdots ,q_{nj}\right)}^{\prime } \end{array}$$

represents a set of relative judgments regarding the quantity levels of all DMUs under the common valuation system determined by DMU-*j*’s optimal DEA weights.

Since each column corresponds to one endogenous evaluation criterion and all columns are generated under different DEA weight systems, the quantity matrix can be interpreted as a collection of pairwise quantity judgments under multiple criteria.

Consequently, the reciprocal quantity matrix possesses a structural similarity to the pairwise comparison matrices employed in the Analytic Hierarchy Process (AHP). The extraction of the principal eigenvector therefore does not introduce an external aggregation rule but follows naturally from the reciprocal comparison structure implied by the quantity decomposition.

However, unlike conventional AHP judgment matrices constructed from subjective expert comparisons, the reciprocal matrices in this study are generated endogenously from DEA cross-efficiency relationships. The comparison information is therefore data-driven in the sense that it originates from observed input-output data and DEA optimization rather than external preference judgments. Nevertheless, the interpretation of these matrices remains different from traditional AHP preference matrices: they represent relative production-position and valuation relationships among DMUs rather than subjective priorities assigned by decision makers.

**Remark 2.** The present study does not propose a new aggregation principle. Instead, it demonstrates that the quantity matrix derived from cross-efficiency information satisfies the mathematical properties required for an AHP-type reciprocal comparison matrix. Therefore, the use of the principal eigenvector follows directly from the established theory of reciprocal matrix aggregation

Since both *Q* and *P* are positive reciprocal matrices, the Perron–Frobenius theorem guarantees the existence of a unique positive principal eigenvector.

**Proposition 4.** *The quantity matrix Q possesses a unique positive principal eigenvector.*

**Proof.** Since $q_{ij}>\text{0}$, for all *i*, *j*, matrix *Q* is strictly positive. According to the Perron–Frobenius theorem, there exists a unique largest eigenvalue $\lambda_{\text{max}}^{Q}$ associated with a strictly positive eigenvector


$$\begin{array}{c} v^{Q}={\left(v_{\text{1}}^{Q},v_{\text{2}}^{Q},\cdots ,v_{n}^{Q}\right)}^{\prime } \end{array}$$

such that


$$\begin{array}{c} Qv^{Q}=\lambda_{\text{max}}^{Q}v^{Q} \end{array}$$

The eigenvector is unique up to a positive scalar multiple.

To obtain a comparable index, define the normalized vector:


$$\begin{array}{c} \begin{array}{c} QI=\frac{v^{Q}}{1^{\prime }v^{Q}} \end{array} \end{array}$$ (10)

where


$$\begin{array}{c} 1=(\text{1},\text{1},\cdots ,\text{1}{)}^{\prime } \end{array}$$

Thus,


$$\begin{array}{c} QI={\left({QI}_{\text{1}},{QI}_{\text{2}},\cdots ,{QI}_{n}\right)}^{\prime } \end{array}$$

and each element is given by ${QI}_{i}=\frac{v_{i}^{Q}}{\sum_{k=\text{1}}^{n}v_{k}^{Q}}$ for i=1,2,⋯,n.

The quantity index ${QI}_{i}$ measures the relative production position of DMU *i* within the cross efficiency system.

Similarly, the price matrix *P* admits a unique positive principal eigenvector:


$$\begin{array}{c} v^{P}={\left(v_{\text{1}}^{P},v_{\text{2}}^{P},\cdots ,v_{n}^{P}\right)}^{\prime } \end{array}$$

satisfying


$$\begin{array}{c} Pv^{P}=\lambda_{\text{max}}^{P}v^{P} \end{array}$$

Define the normalized price index vector as:


$$\begin{array}{c} \begin{array}{c} PI=\frac{v^{P}}{1^{\prime }v^{P}} \end{array} \end{array}$$ (11)

Thus,


$$\begin{array}{c} PI={\left({PI}_{\text{1}},{PI}_{\text{2}},\cdots ,{PI}_{n}\right)}^{\prime } \end{array}$$

where ${PI}_{i}=\frac{v_{i}^{P}}{\sum_{k=\text{1}}^{n}v_{k}^{P}}$ for i=1,2,⋯,n.

The price index ${PI}_{i}$ reflects the relative valuation structure of DMU *i* implied by the cross-efficiency system.

The quantity index captures differences in production positioning across DMUs, while the price index reflects differences in valuation structures generated by alternative DEA weight systems. Together, they provide a dual decomposition of DEA self-efficiency within the cross-efficiency framework.

### Interpretation and scope of the framework

The proposed framework should therefore be interpreted as a decomposition framework for DEA self-efficiency rather than a replacement for existing cross-efficiency evaluation methods. Existing cross-efficiency models aim primarily at improving discrimination, reducing weight flexibility, or obtaining complete rankings among DMUs. In contrast, the present framework focuses on extracting additional structural information from the cross-efficiency matrix after it has been generated.

These two perspectives are complementary rather than competing. Cross-efficiency models provide the relational evaluation system, whereas the proposed decomposition framework investigates how such relational information can be further interpreted through quantity and valuation dimensions. Therefore, the contribution of this study lies in extending the analytical use of cross-efficiency matrices rather than modifying their original evaluation purpose.

Unlike conventional cross-efficiency studies that focus primarily on ranking and discrimination, the present framework aims to reveal the structural composition underlying DEA self-efficiency formation. Specifically, DEA self-efficiency is interpreted as the combined outcome of two complementary mechanisms:

A Production-position effects captured by the quantity index;
B Valuation effects captured by the price index.

Accordingly, the cross-efficiency matrix is transformed from a ranking device into an information structure capable of explaining efficiency formation.

Remark 3. Hierarchical interpretation of the price comparison matrix. The reciprocal structure of the price comparison matrix *P* provides an additional interpretation from the perspective of pairwise comparison systems. Since $p_{ij}=p_{ji}^{-\text{1}}$, the element $p_{ij}\;$represents the relative valuation intensity of DMU *i* with respect to DMU *j* within the DEA-generated valuation system. Therefore, $p_{ij}>\text{1}$ indicates that DMU *i* exhibits a stronger relative valuation position than DMU *j*, whereas $p_{ij}<\text{1}$ indicates the opposite relationship. When $p_{ij}=\text{1}$, the two DMUs occupy equivalent valuation positions within the corresponding comparison system.

However, this interpretation should be distinguished from preference relationships in conventional decision-making contexts. Unlike AHP judgment matrices, where pairwise comparisons usually reflect subjective preferences or expert opinions, the elements of *P* are generated endogenously from DEA cross-efficiency evaluations. Therefore, a higher relative valuation does not imply that one DMU is intrinsically preferred to another, nor does it directly indicate higher efficiency. Instead, it reflects the relative strength of the valuation structure induced by DEA multipliers.

In this sense, the matrix *P* can be viewed as a data-driven reciprocal comparison system embedded within the DEA framework. Its hierarchical interpretation provides a bridge between DEA cross-efficiency analysis and AHP-type pairwise comparison structures while maintaining the data-driven and optimization-based characteristics of DEA.

It is important to reiterate that the framework neither modifies the underlying DEA optimization model nor resolves the multiplicity of optimal DEA weights. Its contribution lies in providing a structural interpretation of DEA efficiency through quantity-related and price-related decomposition, thereby establishing a theoretical foundation for subsequent analyses of technological differences, scale efficiency, and valuation structures.

The quantity index and price index should be interpreted as relative measures within a given technological framework. Their values are therefore technology-dependent rather than technology-free. Consequently, variations in quantity and price indices across alternative DEA technologies may provide additional information regarding technological differences. This observation extends the applicability of the proposed decomposition framework beyond efficiency interpretation and motivates the subsequent analysis of technology-induced quantity and price effects presented in Section 5.

## Empirical illustration

The empirical analysis presented in this section is intended primarily as an illustrative demonstration of the proposed framework rather than a benchmarking exercise designed to establish empirical superiority over existing cross-efficiency methods. The quantity–price decomposition developed in this study constitutes an analytical framework for understanding the formation mechanism of DEA self-efficiency. Its contribution lies in revealing and interpreting the structural information embedded in cross-efficiency matrices, rather than proposing a new optimization algorithm whose value depends on extensive statistical testing across multiple datasets.

For this reason, a classical dataset consisting of eighteen Chinese cities is employed as the empirical example. This dataset has been repeatedly used in the DEA literature and has become a well-recognized benchmark because its efficiency characteristics are transparent and its relatively small scale allows the complete quantity and valuation structures implied by the proposed decomposition to be clearly displayed and interpreted. The use of a simple and familiar dataset is therefore particularly suitable for demonstrating the computational feasibility and interpretive capability of the proposed framework. Employing a larger and more complex dataset would not materially alter the theoretical arguments developed in this paper and might instead obscure the underlying decomposition mechanisms that the study seeks to reveal.

Accordingly, the empirical analysis should be understood as a proof-of-concept illustration that demonstrates how quantity effects and valuation effects can be extracted from cross-efficiency information and how different production technologies may influence the formation of DEA self-efficiency.

### DMU and indicator system

To illustrate the proposed quantity–price decomposition framework, this study examines the relative economic performance of eighteen Chinese cities in 1989, including thirteen coastal open cities and five special economic zones. This dataset has been previously investigated by Zhu [68], Premachandra [69], and Cinca et al. [70], thereby providing a well-established benchmark for evaluating the proposed methodology.

Each decision-making unit (DMU) is characterized by two input indicators and three output indicators. The original data are reported in Table 1.

**Table 1:** Input and output indicators of the DMUs.

DMU	Input 1	Input 2	Output 1	Output 2	Output 3
1	2874.8	16738	160.89	80800	5092
2	946.3	691	21.14	18172	6563
3	6854.0	43024	375.25	144530	2437
4	2305.1	10815	176.68	70318	3145
5	1010.3	2099	102.12	55419	1225
6	282.3	757	59.17	27422	246
7	17478.6	116900	1029.09	351390	14604
8	661.8	2024	30.07	23550	1126
9	1544.2	3218	160.58	59406	2230
10	428.4	574	53.69	47504	430
11	6228.1	29842	258.09	151356	4649
12	697.7	3394	38.02	45336	1555
13	106.4	367	7.07	8236	121
14	4539.3	45809	116.46	56135	956
15	957.8	16947	29.20	17554	231
16	1209.2	15741	65.36	62341	618
17	972.4	23822	54.52	25203	513
18	2192.0	10943	25.24	40267	895

The purpose of this empirical application is not to revisit the efficiency ranking of Chinese cities itself, which has already been extensively discussed in the literature, but rather to demonstrate how the cross-efficiency matrix can be decomposed into complementary quantity and valuation dimensions and how these dimensions vary under alternative technological assumptions.

### Computational procedures

The empirical implementation proceeds in four steps:

**Step 1. Generation of cross-efficiency matrices**

Following Doyle and Green [21], two-stage aggressive and benevolent cross-efficiency models are employed. Under the CRS assumption, both aggressive and benevolent formulations are considered. Under the VRS assumption, the orientation of the model plays a crucial role in the construction of the cross-efficiency matrix. Accordingly, four alternative specifications are considered, namely the input-oriented aggressive model, the input-oriented benevolent model, the output-oriented aggressive model, and the output-oriented benevolent model.

Based on these specifications, six cross-efficiency matrices are consequently generated, including the CRS aggressive matrix, the CRS benevolent matrix, the VRS input-oriented aggressive matrix, the VRS input-oriented benevolent matrix, the VRS output-oriented aggressive matrix, and the VRS output-oriented benevolent matrix, thereby providing a comprehensive framework that captures differences in returns-to-scale assumptions and orientation settings.

The first-stage optimization determines DEA self-efficiency scores and optimal weight schemes, whereas the second-stage optimization determines peer-appraisal weights according to aggressive or benevolent objectives. To avoid the possibility of negative cross-efficiency values caused by the sign of the VRS free variable, the modified formulation proposed by Lim et al. [38] is adopted throughout the analysis.

The resulting cross-efficiency matrices are reported in S1 Table.

**Step 2. Construction of comparison matrices**

For each cross-efficiency matrix, the corresponding quantity comparison matrix and price comparison matrix are constructed according to Equations (4)–(7) developed in Section 3. The calculated quantity matrix and price matrix are shown in S2 and S3 Tables.

**Step 3. Eigenvector aggregation**

The principal right eigenvectors associated with the maximum eigenvalues are then extracted and normalized so that their elements sum to unity. The normalized eigenvectors of the quantity matrices constitute the QIs, whereas those of the price matrices constitute the PIs.

**Step 4. Numerical adjustment**

Under the VRS Input Aggressive model, all off-diagonal elements in the thirteenth row of the cross-efficiency matrix are equal to zero. Since the subsequent construction of reciprocal comparison matrices requires strictly positive entries, all off-diagonal elements in this row are replaced by ${\text{1}\text{0}}^{-\text{5}}$.

Because this perturbation is several orders of magnitude smaller than the observed cross-efficiency values, its influence on the estimated indices is negligible.

### Quantity index: production-position effects

Applying the decomposition procedure developed in Section 3, the principal eigenvectors of the reciprocal quantity matrices are extracted to obtain the Quantity Indices (QIs), which are reported in Table 2.

**Table 2:** QIs derived from six cross-efficiency matrices.

DMU	${QI}_{A}^{CRS}$	${QI}_{B}^{CRS}$	${QI}_{IA}^{VRS}$	${QI}_{IB}^{VRS}$	${QI}_{OA}^{VRS}$	${QI}_{OB}^{VRS}$
1	0.0280	0.0472	0.0043	0.0652	9.4747E-06	0.0669
2	0.3335	0.1117	0.0098	0.0683	6.5961E-05	0.0587
3	0.0168	0.0287	0.0037	0.0544	8.1869E-06	0.0613
4	0.0318	0.0527	0.0040	0.0625	9.1811E-06	0.0655
5	0.0426	0.0647	0.0055	0.0688	1.4499E-05	0.0693
6	0.1078	0.1099	0.0072	0.0709	2.4315E-05	0.0665
7	0.0212	0.0364	0.0146	0.0614	2.8392E-05	0.0670
8	0.0312	0.0491	0.0029	0.0490	5.8514E-06	0.0375
9	0.0620	0.0992	0.0067	0.0674	1.8780E-05	0.0686
10	0.1458	0.0980	0.0190	0.0743	3.9850E-05	0.0711
11	0.0181	0.0300	0.0052	0.0599	1.1318E-05	0.0653
12	0.0459	0.0756	0.0037	0.0661	8.9157E-06	0.0656
13	0.0447	0.0711	0.9039	0.0602	9.9973E-01	0.0450
14	0.0078	0.0137	0.0013	0.0214	3.7807E-06	0.0314
15	0.0098	0.0179	0.0012	0.0222	2.4977E-06	0.0224
16	0.0249	0.0443	0.0035	0.0645	7.7868E-06	0.0673
17	0.0171	0.0319	0.0020	0.0377	4.0925E-06	0.0376
18	0.0109	0.0179	0.0015	0.0256	4.1784E-06	0.0333
$\lambda_{\text{max}}$	18.3131	18.1117	19.6878	18.4679	18.6330	18.1222

Note: $\begin{array}{c} \begin{array}{c} {QI}_{A}^{CRS}:\text{ the QI derived from the CRS aggressive cross efficiency matrix}\\ {QI}_{B}^{CRS}:\text{ the QI derived from the CRS benevolent cross efficiency matrix}\\ {QI}_{IA}^{VRS}:\text{ the QI derived from the VRS input}-\text{oriented aggressive cross efficiency matrix}\\ {QI}_{IB}^{VRS}:\text{ the QI derived from the VRS output}-\text{oriented benevolent cross efficiency matrix}\\ {QI}_{OA}^{VRS}:\text{ the QI derived from the VRS output}-\text{oriented aggressive cross efficiency matrix}\\ {QI}_{OB}^{VRS}:\text{ the QI derived from the VRS output}-\text{oriented benevolent cross efficiency matrix} \end{array} \end{array}$

The corresponding Pearson correlation coefficients among the six QIs are presented in Table 3.

**Table 3:** Pearson correlation matrix of the QIs.

		${QI}_{A}^{CRS}$	${QI}_{B}^{CRS}$	${QI}_{IA}^{VRS}$	${QI}_{IB}^{VRS}$	${QI}_{OA}^{VRS}$	${QI}_{OB}^{VRS}$
${QI}_{A}^{CRS}$	Pearson Correlation	1	0.7452	−0.0233	0.4441	−0.0347	0.2664
	Sig. (2-tailed)		0.0004	0.9268	0.0649	0.8914	0.2852
	N	18	18	18	18	18	18
${QI}_{B}^{CRS}$	Pearson Correlation	0.7452	1	0.1326	0.7599	0.1204	0.5588
	Sig. (2-tailed)	0.0004		0.5999	0.0003	0.6343	0.0159
	N	18	18	18	18	18	18
${QI}_{IA}^{VRS}$	Pearson Correlation	−0.0233	0.1326	1	0.0811	0.9998	−0.1516
	Sig. (2-tailed)	0.9268	0.5999		0.7491	0.0000	0.5480
	N	18	18	18	18	18	18
${QI}_{IB}^{VRS}$	Pearson Correlation	0.4441	0.7599	0.0811	1	0.0677	0.9197
	Sig. (2-tailed)	0.0649	0.0003	0.7491		0.7895	0.0000
	N	18	18	18	18	18	18
${QI}_{OA}^{VRS}$	Pearson Correlation	−0.0347	0.1204	0.9998	0.0677	1	−0.1641
	Sig. (2-tailed)	0.8914	0.6343	0.0000	0.7895		0.5153
	N	18	18	18	18	18	18
${QI}_{OB}^{VRS}$	Pearson Correlation	0.2664	0.5588	−0.1516	0.9197	−0.1641	1
	Sig. (2-tailed)	0.2852	0.0159	0.5480	0.0000	0.5153	
	N	18	18	18	18	18	18

Several observations emerge. First, the two CRS specifications exhibit a relatively strong and statistically significant positive correlation (0.7452, p<0.01). This result indicates that, under a common CRS technology, the dominant production-position information extracted from cross-efficiency matrices remains reasonably stable across alternative secondary objectives. Although aggressive and benevolent formulations generate different peer-appraisal weights, these differences do not substantially alter the principal production-position patterns reflected by the QIs.

Second, a remarkably different pattern emerges under the aggressive VRS specifications. The Quantity Indices obtained from the Input Aggressive and Output Aggressive models are almost perfectly correlated (0.9998, p<0.001), indicating that orientation changes have virtually no effect on the dominant production-position information under aggressive peer-appraisal objectives. More importantly, both aggressive formulations exhibit an extreme concentration phenomenon. Under the Input Aggressive specification, DMU13 alone accounts for approximately 90.39% of the normalized Quantity Index, while under the Output Aggressive specification its share further increases to approximately 99.97%.

This concentration should not be interpreted as implying that a single DMU completely determines the underlying production structure. Rather, it suggests that the reciprocal quantity comparison system identifies one observation as overwhelmingly dominant in the extracted production-position representation under aggressive VRS technologies. Consequently, the informational content summarized by the principal eigenvector becomes highly concentrated on a single reference unit.

Third, the benevolent formulations display a substantially different pattern. The Quantity Indices derived from the Input Benevolent and Output Benevolent VRS models are strongly and significantly correlated (0.9197, p<0.001), yet the resulting distributions remain relatively balanced across DMUs and do not exhibit extreme concentration on any single observation. Hence, benevolent specifications appear to preserve a more diversified production-position representation.

Fourth, the correlation structure reveals considerable heterogeneity across alternative DEA specifications. Several pairwise correlations are weak, statistically insignificant, or even slightly negative. In particular, the correlations between the CRS Aggressive Quantity Index and the two VRS Aggressive Quantity Indices are virtually zero (−0.0233 and −0.0347, respectively). By contrast, the CRS Benevolent Quantity Index remains substantially correlated with both VRS Benevolent measures (0.7599 and 0.5588).

Taken together, these results indicate that the production-position information extracted from cross-efficiency matrices is highly sensitive to the adopted DEA specification. Alternative assumptions regarding returns to scale, orientation, and secondary objectives may lead to markedly different Quantity Indices even when they are applied to the same observed dataset. Consequently, the QI should be interpreted as a technology-conditioned representation of dominant production-position information rather than as an invariant characteristic of the underlying production system.

Remark 4. The term “Quantity Index” does not imply a complete purification of quantity information. Since every cross-efficiency element is jointly determined by observed input-output quantities and endogenous shadow prices, residual valuation influences inevitably remain. The QI should therefore be interpreted as a quantity-dominated representation that emphasizes production-position information without completely eliminating its interaction with valuation effects.

### Price index: Valuation effects

Because Proposition 3 establishes that the quantity and price comparison matrices share identical eigenvalues, differences between the Quantity Index (QI) and the Price Index (PI) arise entirely from their eigenvector structures. Consequently, the PI contains information that is complementary to, rather than redundant with, the QI and provides an alternative perspective for quantifying the valuation-related information embedded in DEA self-efficiency differences.

Table 4 reports the PIs derived from the six cross-efficiency matrices, and Table 5 presents the corresponding descriptive statistics.

**Table 4:** PIs derived from six cross-efficiency matrices.

DMU	CRS		VRS			
	Aggressive	Benevolent	Input Aggressive	Input Benevolent	Output Aggressive	Output Benevolent
1	0.0614	0.0560	0.0732	0.0605	0.0691	0.0538
2	0.0122	0.0507	0.0498	0.0625	0.0122	0.0649
3	0.0605	0.0544	0.0829	0.0651	0.0740	0.0534
4	0.0577	0.0536	0.0754	0.0594	0.0698	0.0531
5	0.0568	0.0555	0.0554	0.0574	0.0475	0.0520
6	0.0348	0.0514	0.0491	0.0578	0.0280	0.0550
7	0.0614	0.0554	0.0275	0.0653	0.0250	0.0541
8	0.0593	0.0567	0.0555	0.0430	0.0680	0.0538
9	0.0398	0.0403	0.0489	0.0586	0.0359	0.0526
10	0.0260	0.0582	0.0272	0.0540	0.0172	0.0511
11	0.0606	0.0565	0.0651	0.0668	0.0581	0.0554
12	0.0627	0.0585	0.0765	0.0552	0.0742	0.0537
13	0.0621	0.0595	0.0005	0.0785	0.0000	0.0852
14	0.0654	0.0569	0.0650	0.0475	0.0826	0.0535
15	0.0724	0.0592	0.0585	0.0384	0.0844	0.0510
16	0.0709	0.0602	0.0898	0.0616	0.0868	0.0534
17	0.0700	0.0554	0.0550	0.0347	0.0843	0.0486
18	0.0660	0.0618	0.0449	0.0336	0.0828	0.0553

**Table 5:** Descriptive statistics of the PIs.

Model	Minimum	Maximum	Mean	Standard Deviation	Variance
CRS Aggressive	0.0122	0.0724	0.0556	0.0164	2.7049E-04
CRS Benevolent	0.0403	0.0618	0.0556	0.0048	2.2827E-05
VRS Input Aggressive	0.0005	0.0898	0.0556	0.0218	4.7696E-04
VRS Input Benevolent	0.0336	0.0785	0.0556	0.0119	1.4218E-04
VRS Output Aggressive	0.0000	0.0868	0.0556	0.0286	8.1912E-04
VRS Output Benevolent	0.0486	0.0852	0.0556	0.0081	6.5185E-05

Several findings deserve attention. First, the estimated Price Indices generally exhibit a more balanced distribution across DMUs than the corresponding Quantity Indices. Although the VRS Output Aggressive specification produces the largest dispersion among the six formulations, with a standard deviation of 0.0286 and a variance of ($\text{8}.\text{1}\text{9}\text{1}\text{2}\times {\text{1}\text{0}}^{-\text{4}}$), the reciprocal price comparison system does not collapse into an extreme single-DMU-dominated pattern. Hence, valuation-dominated information remains comparatively diversified, even under the most heterogeneous specification.

Second, noticeable differences exist between aggressive and benevolent formulations. The benevolent specifications generate relatively concentrated distributions with substantially smaller variances, whereas aggressive formulations, particularly under VRS technologies, produce much greater heterogeneity in the reciprocal Price Indices. This result indicates that alternative peer-appraisal objectives induce different endogenous revaluation environments and therefore alter the valuation-dominated representations extracted from cross-efficiency matrices. Consequently, the valuation-related information embedded in DEA self-efficiency differences is highly dependent on the underlying behavioral assumptions adopted by the cross-efficiency models.

Third, the relatively large dispersion observed under the VRS Output Aggressive specification should not be interpreted as valuation instability in the conventional sensitivity-analysis sense. Rather, it reflects the fact that admissible DEA weighting schemes may assign substantially different shadow valuations to identical or comparable production performances. In other words, aggressive peer-appraisal objectives lead to a more heterogeneous revaluation structure without necessarily producing valuation dominance. The result highlights the substantial flexibility of endogenous DEA weighting systems and demonstrates that alternative weighting environments may generate markedly different valuation-dominated representations even when the underlying production data remain unchanged.

A particularly striking result emerges when the PI is compared with the QI under the aggressive VRS formulations. Under the Output Aggressive model, the Quantity Index becomes almost completely concentrated on DMU13, whose normalized weight reaches approximately 0.9997. By contrast, the corresponding Price Index assigned to DMU-13 is virtually zero. A similar, though less extreme, pattern is also observed under the Input Aggressive specification, where DMU13 accounts for approximately 90.39% of the Quantity Index but receives a Price Index weight of only 0.0005.

This evidence suggests that dominance in the quantity-dominated representation does not imply dominance in the valuation-dominated representation and may even coexist with near-complete valuation irrelevance in the reciprocal price comparison system. The result illustrates that the two informational dimensions embedded in cross-efficiency matrices may evolve in fundamentally different directions under alternative technological and behavioral assumptions. Consequently, a DMU occupying an overwhelmingly dominant position in the quantity comparison system does not necessarily exert a comparable influence on the valuation comparison system.

This divergence has important theoretical implications. The Quantity Index and the Price Index characterize two complementary and predominantly distinct informational dimensions embedded in DEA self-efficiency differences. The former is primarily associated with production-position information extracted from reciprocal quantity comparisons, whereas the latter is primarily associated with endogenous revaluation information implied by DEA weighting systems. Because both indices remain partially coupled through the underlying cross-efficiency elements, the proposed decomposition should be interpreted as a dominant-information decomposition rather than a strict orthogonal separation between quantity and valuation effects.

Accordingly, the Price Index should not be interpreted as a robustness measure in the conventional DEA sensitivity-analysis sense. Nor should it be regarded as a pure measure of valuation effects. Instead, it provides an additional explanatory dimension for quantifying the valuation-dominated information embedded in DEA self-efficiency differences and for understanding how alternative DEA weighting systems generate different revaluation environments under a given production technology. Because residual quantity influences cannot be completely eliminated, the PI should be interpreted as a valuation-dominated, technology-conditioned, and partially quantity-dependent representation that complements the Quantity Index in explaining observed differences in DEA self-efficiency.

Remark 5. The term “Price Index” does not imply that the proposed index provides a complete purification of valuation information. Every cross-efficiency element is jointly determined by observed input-output quantities and endogenous DEA shadow prices. The reciprocal price comparison matrix therefore isolates valuation-related information only in a dominant sense. Under a fixed production technology, the PI is predominantly driven by differences in endogenous valuation systems and revaluation structures, while residual quantity influences inevitably remain. The proposed decomposition should therefore be interpreted as a dominant-information decomposition in which the QI and PI respectively emphasize quantity-related and valuation-related information without completely eliminating their mutual dependence.

### Technology-conditioned quantity and valuation effects

The preceding analyses demonstrate that the Quantity Index and the Price Index capture two complementary informational dimensions embedded in cross-efficiency systems. The QI predominantly summarizes production-position information, whereas the PI predominantly summarizes endogenous valuation information. The central question is therefore why both indices may change substantially when alternative DEA specifications are adopted.

The answer lies in the fact that changes in DEA specifications simultaneously modify two interconnected structures.

The first structure is the production possibility set. Altering returns-to-scale assumptions or orientation specifications reshapes the attainable production frontier and changes the relative positions of DMUs within the production space. Because the reciprocal quantity comparison matrix is constructed from relative production comparisons, modifications of the production possibility set may substantially alter the Quantity Index. The extreme concentration of the QI on DMU13 under aggressive VRS specifications illustrates that changes in technology descriptions may induce profound reorganizations of the dominant production-position information extracted from cross-efficiency matrices.

The second structure is the admissible valuation system. Every cross-efficiency model generates a feasible set of endogenous shadow prices and peer-appraisal weights. Changes in orientation, returns-to-scale assumptions, and secondary-goal formulations modify the admissible weighting space and consequently alter the endogenous revaluation environment embodied in the reciprocal price comparison matrix. The substantial heterogeneity observed among the PIs, particularly under aggressive VRS specifications, demonstrates that alternative technologies may induce markedly different valuation structures even when the observed input-output data remain unchanged.

Importantly, these two channels are not mechanically synchronized. A technological modification that substantially changes production positioning may generate only limited changes in endogenous valuation structures, and vice versa. The empirical results under the aggressive VRS specifications provide a striking illustration. Under these models, the Quantity Index becomes almost completely concentrated on DMU13, indicating an overwhelming dominance in the production-position representation. However, the corresponding Price Index assigned to the same DMU is nearly zero, implying that the endogenous valuation system does not attribute a comparable degree of valuation importance to that observation.

This divergence has important implications for interpreting DEA self-efficiency. Dominance in production space does not necessarily imply dominance in valuation space. Similarly, changes in efficiency estimates induced by alternative DEA specifications cannot generally be attributed solely to frontier repositioning or solely to weighting flexibility. Instead, DEA self-efficiency should be understood as the outcome of a structured interaction between production-position relationships and endogenous valuation systems.

Accordingly, the proposed decomposition framework provides an operational lens for disentangling these two sources of variation. By jointly examining the QI and PI, researchers can identify whether observed changes in self-efficiency primarily originate from changes in production positioning, changes in valuation structures, or from the interaction of both channels. The framework therefore offers a richer interpretation of model dependence and provides additional insights into the formation mechanism of DEA self-efficiency and the interpretation of scale efficiency under alternative technologies.

Finally, because the present study is primarily methodological, the empirical illustration is intended to demonstrate the operational feasibility and interpretive capability of the proposed framework rather than to establish empirical superiority of any particular DEA specification. The principal contribution lies in showing how production-position effects and valuation effects can be extracted from cross-efficiency information and how alternative technologies simultaneously reshape these two informational dimensions.

## Applications of the quantity–price decomposition framework

The quantity–price decomposition developed in this study provides information that is not directly available from conventional DEA efficiency scores or from cross-efficiency rankings alone. By separating production-position effects from endogenous valuation effects, the proposed framework offers additional analytical tools for interpreting DEA results and understanding the mechanisms through which efficiency evaluations are generated.

This section illustrates two immediate applications of the proposed framework. The first concerns the interpretation of scale efficiency through the Quantity Index (QI). The second concerns the characterization of endogenous valuation structures through the Price Index (PI).

### Quantity index and a refined interpretation of scale efficiency

#### Limitations of conventional scale efficiency.

Scale efficiency is traditionally defined as the ratio between technical efficiency under constant returns to scale (CRS) and technical efficiency under variable returns to scale (VRS):


$$\begin{array}{c} \begin{array}{c} {SE}_{i}=\frac{{TE}_{i}^{CRS}}{{TE}_{i}^{VRS}} \end{array} \end{array}$$ (12)

where ${TE}_{i}^{CRS}$ and ${TE}_{i}^{VRS}$ denote the efficiency scores obtained under constant and variable returns-to-scale technologies, respectively.

Under this framework, deviations of $SE_{i}$ from unity are interpreted as evidence that a DMU operates at a non-optimal scale (Banker et al., 1984). The measure has become one of the most widely used indicators in DEA applications.

However, the conventional interpretation implicitly assumes that the difference between CRS and VRS efficiencies is entirely attributable to scale effects. This assumption deserves further scrutiny. Moving from a CRS technology to a VRS technology simultaneously changes the production possibility set, modifies peer relationships, and alters the endogenous valuation systems generated by DEA models. Consequently, observed differences between ${TE}^{CRS}$ and ${TE}^{VRS}$ may arise not only from scale effects but also from changes in production positions and valuation structures.

Because conventional scale efficiency provides only a numerical ratio between two efficiency scores, it offers little information regarding the mechanisms through which these differences are generated. The quantity–price decomposition developed in this study provides an opportunity to examine this issue from a new perspective.

#### Quantity-based decomposition of scale efficiency.

The conventional SE confounds two effects: a pure scale effect and a technology-induced repositioning effect. By comparing QIs under CRS and VRS, we can isolate the latter, as QI specifically captures production-position information. Therefore, the ratio of QIs serves as an indicator of this repositioning effect, offering a refined perspective on the sources of SE differences.

The Quantity Index characterizes the relative production positions of DMUs embedded within the cross-efficiency information system. Let $QI_{i}^{CRS}$ and $QI_{i}^{VRS}$ denote the Quantity Indices obtained under CRS and VRS technologies, respectively. The relative change in production position can be measured by


$$\begin{array}{c} \begin{array}{c} {SE}_{i}^{Q}=\frac{{QI}_{i}^{CRS}}{{QI}_{i}^{VRS}} \end{array} \end{array}$$ (13)

where ${QI}_{i}^{CRS}$ and ${QI}_{i}^{VRS}$ denote the Quantity Indices obtained under CRS and VRS technologies, respectively.

Unlike conventional scale efficiency, ${SE}_{i}^{Q}$ does not measure productive efficiency itself. Instead, it measures the degree to which alternative technological assumptions reposition a DMU within the peer-evaluation structure.

If ${SE}_{i}^{Q}\approx \text{1}$, the relative production position of DMU *i* remains largely unchanged under alternative technologies, suggesting that the difference between ${TE}_{i}^{CRS}$ and ${TE}_{i}^{VRS}$ may be interpreted primarily as a conventional scale effect.

Conversely, substantial deviations of ${SE}_{i}^{Q}$ from unity indicate that technology-induced repositioning effects contribute significantly to the observed scale-efficiency differences.

Therefore, conventional scale efficiency may be viewed as a composite outcome generated by at least two mechanisms: the traditional scale effect associated with non-optimal scale operations; and technology-induced production repositioning effects captured by the Quantity Index.

The proposed quantity-based indicator provides an additional layer of information that is unavailable from conventional DEA scale-efficiency measures.

#### Empirical illustration.

To illustrate the usefulness of the proposed framework, the quantity-based scale indicator was calculated for the Chinese-city dataset. The results are reported in Table 6 and Table 7.

**Table 6:** Input-oriented conventional and production-position-based scale efficiency.

DMU	${TE}^{CRS}$	${TE}_{I}^{VRS}$	${SE}_{I}$	${QI}^{CRS}$	${QI}_{IB}^{VRS}$	${SE}_{I}^{Q}$
1	0.4691	1.0000	0.4691	0.0472	0.0652	0.7241
2	1.0000	1.0000	1.0000	0.1117	0.0683	1.6351
3	0.2779	0.8656	0.2779	0.0287	0.0544	0.5279
4	0.5022	0.9421	0.5022	0.0527	0.0625	0.8431
5	0.6311	1.0000	0.6311	0.0647	0.0688	0.9413
6	1.0000	1.0000	1.0000	0.1099	0.0709	1.5498
7	0.3580	1.0000	0.3580	0.0364	0.0614	0.5926
8	0.4959	0.5148	0.4959	0.0491	0.0490	1.0018
9	0.6577	1.0000	0.6577	0.0992	0.0674	1.4724
10	1.0000	1.0000	1.0000	0.0980	0.0743	1.3176
11	0.3010	1.0000	0.3010	0.0300	0.0599	0.5002
12	0.7866	0.9202	0.7866	0.0756	0.0661	1.1426
13	0.7514	1.0000	0.7514	0.0711	0.0602	1.1810
14	0.1382	0.2497	0.1382	0.0137	0.0214	0.6407
15	0.1867	0.2040	0.1867	0.0179	0.0222	0.8053
16	0.4704	1.0000	0.4704	0.0443	0.0645	0.6862
17	0.3059	0.3095	0.3059	0.0319	0.0377	0.8477
18	0.1953	0.1967	0.1953	0.0179	0.0256	0.6960

**Table 7:** Output-oriented conventional and production-position-based scale efficiency.

DMU	${TE}^{CRS}$	${TE}_{O}^{VRS}$	${SE}_{O}$	${QI}^{CRS}$	${QI}_{OB}^{VRS}$	${SE}_{O}^{Q}$
1	0.4691	1.0000	0.4691	0.0472	0.0669	0.7058
2	1.0000	1.0000	1.0000	0.1117	0.0587	1.9021
3	0.2779	0.8962	0.3101	0.0287	0.0613	0.4687
4	0.5022	0.9658	0.5200	0.0527	0.0655	0.8056
5	0.6311	1.0000	0.6311	0.0647	0.0693	0.9342
6	1.0000	1.0000	1.0000	0.1099	0.0665	1.6538
7	0.3580	1.0000	0.3580	0.0364	0.0670	0.5430
8	0.4959	0.5566	0.8911	0.0491	0.0375	1.3110
9	0.6577	1.0000	0.6577	0.0992	0.0686	1.4469
10	1.0000	1.0000	1.0000	0.0980	0.0711	1.3782
11	0.3010	1.0000	0.3010	0.0300	0.0653	0.4588
12	0.7866	0.9739	0.8077	0.0756	0.0656	1.1515
13	0.7514	1.0000	0.7514	0.0711	0.0450	1.5822
14	0.1382	0.4620	0.2991	0.0137	0.0314	0.4360
15	0.1867	0.3159	0.5910	0.0179	0.0224	0.7990
16	0.4704	1.0000	0.4704	0.0443	0.0673	0.6581
17	0.3059	0.5006	0.6112	0.0319	0.0376	0.8501
18	0.1953	0.5093	0.3834	0.0179	0.0333	0.5368

The empirical results reveal substantial heterogeneity in technology-induced production repositioning effects across cities. Under the input orientation, only a few DMUs, such as DMU5, DMU8, and DMU15, exhibit relatively similar quantity indices under CRS and VRS technologies, with the corresponding values of (${SE}_{I}^{Q}$) remaining close to unity. For most cities, however, the quantity-based scale indicators deviate considerably from one, indicating that the transition from CRS to VRS technologies substantially alters their relative production positions within the cross-efficiency information system. The output-oriented results display an even stronger pattern, with ($SE_{O}^{Q}$) ranging from 0.4360 to 1.9021, suggesting pronounced technology-induced repositioning effects.

Moreover, the observed repositioning effects are not unidirectional. While some DMUs experience an improvement in their relative production positions after the adoption of VRS technology ($SE^{Q}>\text{1}$), others exhibit a deterioration ($SE^{Q}<\text{1}$). This finding implies that the shift from CRS to VRS not only changes the shape of the production frontier but also reconstructs the relative evaluation relationships among DMUs.

A particularly noteworthy result is that conventional scale efficiency and quantity-based scale efficiency may lead to fundamentally different interpretations. Several DMUs, including DMU2, DMU6, and DMU10, are identified as fully scale efficient according to the conventional measure (SE=1). Nevertheless, their quantity-based scale indicators remain substantially larger than unity, indicating that significant production repositioning effects persist even when conventional scale inefficiency has disappeared. Consequently, variations in DEA scale efficiency should not be interpreted solely as scale effects. Instead, they also embody changes in the relative production positions induced by alternative technological assumptions. By explicitly quantifying this repositioning component, the proposed Quantity Index provides a more refined interpretation of scale efficiency and offers additional insights into the mechanisms underlying the formation of DEA self-efficiency under different production technologies.

### Price index and endogenous valuation structures

One of the most distinctive characteristics of DEA is its endogenous weight determination mechanism. Because DEA efficiency is obtained through weight optimization, different DMUs may employ substantially different weighting schemes when assessing performance. The reciprocal Price Matrix constructed in this study summarizes these revaluation relationships and reveals how observed performances are assessed under alternative peer-generated weighting systems. The corresponding Price Index (PI) therefore provides a quantitative representation of the valuation-dominated information embedded in cross-efficiency matrices.

A relatively high PI indicates that the observed performance of a DMU receives favorable assessments under a broad range of admissible weighting environments and therefore enjoys relatively widespread support from alternative endogenous valuation systems. Conversely, a relatively low PI suggests that favorable assessments of a DMU are sustained only under a relatively limited subset of admissible weighting schemes. Importantly, a low PI should not be interpreted as evidence of weak productive capability. Rather, it indicates limited valuation importance within the endogenous revaluation environment generated by a particular DEA specification.

The empirical evidence reported in Tables 4 and 5 demonstrates that valuation-dominated information generally remains more diversified than quantity-dominated information. Although the VRS Output Aggressive specification produces substantially larger dispersion than the other formulations, the reciprocal price comparison system does not collapse into an extreme single-DMU-dominated pattern. Therefore, alternative DEA specifications may generate markedly different degrees of valuation heterogeneity while preserving comparatively distributed revaluation structures.

Substantial differences nevertheless arise across alternative orientations and secondary-goal formulations, indicating that different DEA specifications generate markedly different endogenous revaluation environments. In particular, aggressive formulations under VRS technologies induce considerably greater heterogeneity in the Price Indices than benevolent formulations, implying that peer-appraisal objectives play an important role in shaping valuation-dominated representations.

Importantly, the PI should not be interpreted as a measure of productive capability. Production-related information is predominantly reflected by the Quantity Index. Instead, the PI provides information regarding how alternative admissible weighting systems evaluate observed production performances and how different DEA specifications generate different revaluation environments. Because residual quantity influences cannot be completely eliminated, the PI should be interpreted as a valuation-dominated, technology-conditioned, and partially quantity-dependent representation of DEA self-efficiency differences.

The empirical results further reveal that quantity-dominated information and valuation-dominated information may become strongly decoupled. Under the aggressive VRS formulations, DMU13 almost completely dominates the Quantity Index while receiving virtually zero Price Index weight. This finding demonstrates that dominance in the quantity comparison system does not necessarily imply broad support within the reciprocal price comparison system. Consequently, production-position information and endogenous revaluation information should be viewed as complementary but only predominantly distinct dimensions of DEA self-efficiency differences.

This observation has important implications for DEA model specification. Conventional model selection in DEA is typically based on theoretical considerations, returns-to-scale assumptions, orientations, or robustness analyses. However, alternative DEA specifications may generate fundamentally different endogenous revaluation environments even when applied to the same production dataset. The proposed Price Index provides an additional diagnostic perspective for examining these differences. Specifications associated with highly concentrated PIs indicate that valuation support is disproportionately allocated to a relatively small subset of DMUs, whereas more evenly distributed PIs suggest broader valuation acceptance across alternative weighting environments.

Therefore, the PI may serve as a useful reference for DEA model selection and specification assessment. It does not identify a universally optimal DEA model, nor should it be interpreted as a formal model-selection criterion. Rather, it provides supplementary information regarding the valuation characteristics generated by competing DEA specifications and enables researchers to evaluate the extent of valuation concentration, heterogeneity, and peer-support diversity implied by alternative weighting environments. When several DEA formulations produce similar self-efficiency results, the PI can offer additional evidence for selecting specifications whose endogenous revaluation structures are more consistent with the analytical objectives of the study.

Taken together, the applications presented above demonstrate that the proposed decomposition framework provides information unavailable from conventional DEA efficiency scores alone. The Quantity Index characterizes quantity-dominated representations associated with production positions and technological assumptions, whereas the Price Index characterizes valuation-dominated representations associated with endogenous revaluation environments. By jointly examining these two complementary informational dimensions, researchers can obtain a richer understanding of DEA self-efficiency differences and a new perspective for interpreting model dependence and evaluating alternative DEA specifications.

## Discussion

The objective of this study is not to rediscover the determinants of DEA efficiency, which are already well established in the DEA literature. It is widely recognized that DEA self-efficiency is jointly determined by observed input-output configurations, production technologies, and endogenous weighting systems. Instead, the present study provides a quantitative and informational perspective on DEA self-efficiency differences by exploiting the informational structure embedded in cross-efficiency matrices. Through the construction of reciprocal Quantity and Price Indices, the proposed framework quantifies the relative influences of quantity-related and valuation-related information and provides additional insights that cannot be directly obtained from conventional DEA efficiency scores. The discussion therefore focuses on three issues: the technology-conditioned and model-dependent nature of DEA self-efficiency, the informational interpretation of cross-efficiency matrices, and the potential applications of the proposed indices.

### DEA self-efficiency as a technology-conditioned and model-dependent measure

The empirical results demonstrate that DEA self-efficiency should be interpreted as a technology-conditioned and model-dependent measure rather than as an invariant representation of productive performance. Different returns-to-scale assumptions, orientations, and secondary-goal formulations generate markedly different Quantity and Price Indices even when they are applied to the same production dataset. This finding reinforces the well-known observation that DEA efficiency scores depend on modeling assumptions, but it further suggests that such dependence extends beyond numerical efficiency values and also affects the informational structures underlying self-efficiency differences.

The proposed decomposition indicates that technological assumptions may simultaneously alter production possibility sets, reshape relative production positions, and generate different endogenous revaluation environments. Consequently, differences among DEA specifications should not necessarily be interpreted as measurement errors or robustness problems. Rather, they reflect alternative informational representations of the same production system generated by different technological and behavioral assumptions. The proposed indices therefore provide a quantitative perspective for examining how these assumptions influence observed self-efficiency differences.

Importantly, the present study does not claim that the proposed Quantity and Price Indices identify previously unknown determinants of DEA efficiency. The determinants themselves are already well understood in DEA theory. Instead, the contribution of the decomposition lies in making the influences of technological assumptions and endogenous weighting systems more transparent and measurable and in providing additional information for interpreting differences among alternative DEA specifications.

### Cross-efficiency matrices as information systems

Existing cross-efficiency studies primarily treat the cross-efficiency matrix as a computational device for peer evaluation, ranking, and consensus building. By contrast, the present study treats the cross-efficiency matrix as an information system containing rich structural information regarding DEA self-efficiency differences.

The reciprocal Quantity and Price Matrices reveal two complementary informational dimensions embedded in cross-efficiency relationships. The Quantity Index predominantly reflects information associated with observed input-output configurations and relative production positions, whereas the Price Index predominantly reflects information associated with endogenous revaluation structures generated by alternative weighting schemes. Because both indices are derived from the same cross-efficiency elements, they remain partially coupled and cannot be interpreted as pure quantity and pure valuation measures.

Accordingly, the proposed framework should be interpreted as a dominant-information decomposition rather than a strict orthogonal separation between quantity and valuation effects. Under a fixed production technology, the QI is primarily driven by differences in production positions and observed input-output configurations, while residual valuation influences remain unavoidable. Similarly, the PI is primarily driven by endogenous revaluation structures, while residual quantity influences cannot be completely eliminated.

Nevertheless, the decomposition succeeds in extracting two complementary informational dimensions that are largely concealed in conventional DEA efficiency measures. The findings therefore extend the role of cross-efficiency matrices from ranking devices to information carriers capable of supporting the quantitative interpretation of DEA self-efficiency differences.

### Applications of the quantity and price indices

The proposed indices have several potential applications that are unavailable from conventional DEA efficiency scores.

First, the indices provide additional information for understanding model dependence. Because alternative DEA specifications generate different quantity-dominated and valuation-dominated representations, researchers can identify whether observed efficiency differences arise primarily from changes in production-position information, changes in endogenous revaluation environments, or the combined effects of both. The decomposition therefore provides a richer interpretation of why different DEA models may produce substantially different efficiency results.

Second, the Quantity Index provides an alternative mechanism for aggregating cross-efficiency information. Existing cross-efficiency studies generally employ arithmetic means, weighted averages, or consensus-based procedures to aggregate peer evaluations. Although these approaches summarize the average evaluations received by each DMU, they largely ignore the reciprocal comparison relationships embedded in cross-efficiency matrices. By contrast, the QI is derived from the principal eigenvector of the reciprocal quantity matrix and simultaneously incorporates all pairwise production-position comparisons contained in the cross-efficiency information system. Consequently, the QI may be interpreted as a structure-preserving aggregation of cross-efficiency information rather than as a simple averaging procedure.

Under a fixed production technology, the QI is predominantly driven by observed input-output configurations and relative production positions, while residual valuation influences remain comparatively small. In this setting, the QI may be viewed as an aggregated representation of the production-position information embedded in the reciprocal comparison system. Therefore, the proposed framework provides an additional perspective for synthesizing cross-efficiency information while preserving the structural relationships among DMUs. The QI values and the corresponding rankings obtained from the proposed aggregation procedure are reported in S4 Table.

The empirical evidence demonstrates that the rankings generated by QI aggregation and conventional mean aggregation need not coincide. Under the CRS and benevolent VRS formulations, the two aggregation schemes often produce broadly similar ordering patterns because the reciprocal quantity structures remain sufficiently diversified. However, substantial discrepancies emerge under the aggressive VRS formulations. In particular, DMU-13 exhibits only modest performance according to conventional mean aggregation but becomes the dominant unit under QI aggregation. This result should not be interpreted as evidence of superior productive performance. Instead, it indicates that the reciprocal quantity comparison system has become highly concentrated and that DMU-13 occupies a structurally dominant position within the production-position network.

Accordingly, conventional mean aggregation and QI aggregation summarize different informational contents embedded in cross-efficiency matrices. The former represents an evaluative aggregation that reflects average peer-appraisal performance, whereas the latter represents a structural aggregation that reflects the relative importance of DMUs within reciprocal production-position relationships. The two aggregation mechanisms need not coincide and may diverge substantially under particular technological and behavioral assumptions. The QI therefore provides not merely another aggregation method but also an additional mechanism for extracting and synthesizing the structural information contained in cross-efficiency systems.

Third, the Price Index may serve as a useful reference for DEA model specification assessment. Conventional model selection in DEA is usually based on theoretical considerations, returns-to-scale assumptions, orientations, and robustness analyses. However, alternative DEA specifications may generate fundamentally different endogenous revaluation environments even when applied to identical production data. The PI provides supplementary information regarding the valuation characteristics implied by competing specifications.

Specifications associated with highly concentrated PIs indicate that valuation support is disproportionately allocated to a relatively small subset of DMUs, whereas more evenly distributed PIs suggest broader valuation acceptance across admissible weighting environments. Consequently, when several DEA specifications produce similar self-efficiency results, the PI may provide additional evidence for selecting specifications whose endogenous revaluation structures are more consistent with the analytical objectives of the study. The PI should therefore be regarded as an auxiliary diagnostic reference for model selection and specification assessment rather than a formal model-selection criterion or an indicator of a universally optimal DEA specification.

Finally, the proposed decomposition provides a new perspective for interpreting technology-induced differences in DEA efficiency measures and identifying extreme reciprocal comparison structures. Traditional scale-efficiency analysis is unable to distinguish between changes arising from relative production positions and changes arising from endogenous valuation structures. By jointly examining the QI and PI, researchers may obtain additional information regarding whether observed efficiency differences are primarily associated with changes in quantity-dominated information or valuation-dominated information, thereby facilitating a more nuanced interpretation of technological assumptions in DEA.

Moreover, the empirical results reveal that a DMU may almost completely dominate the reciprocal quantity comparison system while receiving virtually no support in the reciprocal price comparison system. Such findings indicate that quantity-dominated information and valuation-dominated information may evolve in substantially different directions and therefore provide complementary, although not completely independent, perspectives for interpreting DEA self-efficiency differences.

The proposed Quantity and Price Indices therefore provide complementary informational perspectives for interpreting DEA self-efficiency differences and for examining how technological assumptions and endogenous weighting systems shape the observed characteristics of DEA evaluations.

## Conclusions

### Main findings

This study develops a technology-conditioned quantity-price decomposition framework based on cross-efficiency information. By treating the cross-efficiency matrix as an information system rather than merely a computational device for aggregation and ranking, the proposed framework extracts two complementary informational dimensions embedded in DEA self-efficiency differences, namely quantity-dominated information represented by the Quantity Index (QI) and valuation-dominated information represented by the Price Index (PI).

The theoretical analysis establishes a fundamental duality between reciprocal quantity and price representations. Although the corresponding quantity and price matrices characterize different informational dimensions of cross-efficiency relationships, they possess identical spectral properties and differ only in their associated eigenvector structures. Consequently, quantity-related information and valuation-related information are intrinsically linked while remaining predominantly distinct.

The empirical results further demonstrate that both Quantity Indices and Price Indices are strongly conditioned by technological assumptions and peer-appraisal specifications. Alternative DEA models therefore generate different informational representations of the same production system because they simultaneously alter production possibility sets, relative production positions, and endogenous revaluation environments. Moreover, the responses of quantity-dominated and valuation-dominated information to technological changes are highly asymmetric. Under certain specifications, quantity representations may become highly concentrated and nearly dominated by a single DMU, whereas the corresponding valuation representations remain comparatively diversified.

The empirical analysis also reveals that quantity-dominated information and valuation-dominated information may evolve in substantially different directions. Under the aggressive VRS formulations, a DMU may almost completely dominate the Quantity Index while receiving virtually no Price Index weight. This result indicates that dominance in the reciprocal quantity comparison system does not necessarily imply broad support in the reciprocal price comparison system and illustrates the complementary nature of the two informational dimensions extracted from cross-efficiency matrices.

Consequently, DEA self-efficiency should be interpreted as a technology-conditioned and model-dependent measure whose observed differences reflect the joint influences of production technologies, observed input-output configurations, and endogenous weighting systems rather than as an invariant or uniquely defined representation of productive performance.

### Contributions and implications

The contributions of this study are threefold. First, rather than rediscovering the determinants of DEA efficiency, which are already well established in the DEA literature, the proposed framework makes measurable the influences of technological assumptions and endogenous weighting systems on observed differences in DEA self-efficiency. The study therefore provides a quantitative perspective for examining how alternative DEA specifications generate different efficiency representations.

Second, the study extends the role of cross-efficiency matrices from ranking devices to information systems containing rich structural information. By exploiting the informational organization embedded in reciprocal cross-efficiency relationships, the proposed framework extracts two complementary informational dimensions and develops reciprocal Quantity and Price Indices that provide a dominant-information decomposition of DEA self-efficiency differences.

Third, the proposed indices provide several applications that are unavailable from conventional DEA efficiency scores alone. The decomposition framework offers additional information for interpreting model dependence, assessing alternative DEA specifications, examining technology-induced efficiency differences, and identifying extreme reciprocal comparison structures. In particular, the Price Index may serve as a useful diagnostic reference for evaluating the valuation characteristics implied by competing DEA specifications and may provide supplementary evidence when researchers choose among alternative DEA formulations that generate similar efficiency results.

More generally, the present study suggests that differences among DEA models should not be interpreted merely as issues of computational sensitivity or robustness. Instead, alternative DEA specifications generate different quantity-dominated and valuation-dominated representations of the same production system. The contribution of the present study therefore lies not in eliminating the model dependence of DEA efficiency evaluation but in quantifying, decomposing, and interpreting the informational consequences of such dependence.

### Limitations and future research

Several limitations of the present study should be acknowledged. First, the empirical analysis is conducted using a single cross-sectional dataset. Additional applications involving larger samples, panel datasets, and alternative empirical contexts would help assess the empirical regularities and generalizability of the proposed quantity-price decomposition framework.

Second, although the present study demonstrates that the Quantity Index and Price Index provide useful information for interpreting DEA self-efficiency differences and model dependence, their potential applications in productivity analysis, technological-change decomposition, efficiency dynamics, and performance-monitoring systems remain largely unexplored.

Third, the proposed framework represents a dominant-information decomposition rather than a strict orthogonal separation between quantity and valuation information. Because every cross-efficiency element is jointly determined by observed input-output quantities and endogenous DEA shadow prices, the Quantity Index and the Price Index remain partially coupled. Further theoretical research is needed to investigate whether stronger forms of quantity-price separation can be achieved under alternative reciprocal comparison systems.

Finally, the present framework is developed under conventional cross-efficiency settings. Future studies may extend the decomposition approach to weight-restricted DEA, network DEA, dynamic DEA, cooperative-game-based DEA models, and other complex frontier environments. Examining whether the dual informational properties identified in this study persist in these more general settings may contribute to a broader understanding of the informational structures embedded in DEA efficiency evaluation.

Another promising direction for future research concerns the relationship between DEA cross-efficiency structures and AHP-type reciprocal comparison systems. The present study demonstrates that the reciprocal quantity and price comparison matrices derived from cross-efficiency information possess structural similarities with reciprocal comparison matrices in AHP. This connection suggests several possible extensions. First, future studies may investigate the consistency properties of DEA-induced reciprocal matrices by adapting consistency analysis methods developed in AHP. Second, the relationship between multiple DEA weight systems and group comparison mechanisms in AHP deserves further exploration, since cross-efficiency evaluation can also be viewed as a collection of heterogeneous peer assessments generated from different optimization perspectives. Third, uncertainty extensions, such as fuzzy or interval reciprocal comparison systems, may provide new opportunities for integrating uncertain DEA environments with AHP-based decision analysis.

These directions may contribute to the development of a broader theory of DEA relational evaluation systems, where cross-efficiency matrices are not only used for ranking purposes but also analyzed as structured comparison networks containing richer information.

Ultimately, the present study suggests that conventional DEA efficiency scores contain richer informational content than is commonly recognized. By extracting quantity-related and valuation-related information from cross-efficiency matrices, the proposed framework provides a new quantitative perspective for interpreting DEA self-efficiency differences and offers additional tools for understanding the model dependence and informational characteristics of DEA evaluations.

## Supporting information

S1 Table The cross-efficiency matrix. The appendix contains six cross-efficiency matrices under different orientations and types for both CRS and VRS technologies.
(PDF)

S2 Table The quantity matrix. Corresponding to the different cross-efficiency matrices, the appendix contains six quantity matrices.
(PDF)

S3 Table The price matrix. Corresponding to the different cross-efficiency matrices, the appendix contains six price matrices.
(PDF)

S4 Table Aggregated indicators and corresponding rankings. (PDF)
