# Supplementary material for: Decomposing DEA self-efficiency: A cross-efficiency-based quantity–price framework
Source: PLoS One. 2026 Aug 10;21(8):e0355649. doi: 10.1371/journal.pone.0355649 (PMC13456291; doi:10.1371/journal.pone.0355649)
Supplement: Supplementary file 1 [file pone.0355649.s001.pdf]

Supporting information

S1 Table. The Cross efficiency matrix

S1 Table 1. **The Cross Efficiency Matrix Derived from the CRS Aggressive Model**

DMU	1	2	3	4	5	6	7	8	9	10	11	12	13	14	15	16	17	18
1	0.4691	1.0000	0.2490	0.4608	0.6311	1.0000	0.3064	0.4959	0.5566	1.0000	0.3010	0.7627	0.7251	0.1382	0.1867	0.4651	0.3030	0.1837
2	0.0320	1.0000	0.0060	0.0306	0.0614	0.0342	0.0132	0.0586	0.0730	0.0789	0.0164	0.0482	0.0347	0.0022	0.0014	0.0041	0.0023	0.0086
3	0.4679	1.0000	0.2779	0.5022	0.5857	1.0000	0.3580	0.4143	0.6282	0.6610	0.2727	0.5214	0.4294	0.1360	0.1604	0.2953	0.3059	0.1020
4	0.4679	1.0000	0.2779	0.5022	0.5857	1.0000	0.3580	0.4143	0.6282	0.6610	0.2727	0.5214	0.4294	0.1360	0.1604	0.2953	0.3059	0.1020
5	0.4691	1.0000	0.2490	0.4608	0.6311	1.0000	0.3064	0.4959	0.5566	1.0000	0.3010	0.7627	0.7251	0.1382	0.1867	0.4651	0.3030	0.1837
6	0.1375	0.2567	0.1257	0.2282	0.5888	1.0000	0.1276	0.1948	0.6044	1.0000	0.1211	0.1572	0.2577	0.0380	0.0265	0.0629	0.0355	0.0325
7	0.4679	1.0000	0.2779	0.5022	0.5857	1.0000	0.3580	0.4143	0.6282	0.6610	0.2727	0.5214	0.4294	0.1360	0.1604	0.2953	0.3059	0.1020
8	0.4691	1.0000	0.2490	0.4608	0.6311	1.0000	0.3064	0.4959	0.5566	1.0000	0.3010	0.7627	0.7251	0.1382	0.1867	0.4651	0.3030	0.1837
9	0.1827	1.0000	0.1330	0.2671	0.6291	1.0000	0.1459	0.2647	0.6577	1.0000	0.1421	0.2233	0.2963	0.0413	0.0289	0.0696	0.0395	0.0443
10	0.0583	0.3178	0.0406	0.0786	0.3190	0.4377	0.0363	0.1406	0.2231	1.0000	0.0613	0.1614	0.2712	0.0148	0.0125	0.0479	0.0128	0.0445
11	0.4691	1.0000	0.2490	0.4608	0.6311	1.0000	0.3064	0.4959	0.5566	1.0000	0.3010	0.7627	0.7251	0.1382	0.1867	0.4651	0.3030	0.1837
12	0.4389	1.0000	0.2103	0.4082	0.5822	0.8750	0.2612	0.4896	0.4809	1.0000	0.2835	0.7866	0.7514	0.1236	0.1745	0.4704	0.2696	0.1953
13	0.4389	1.0000	0.2103	0.4082	0.5822	0.8750	0.2612	0.4896	0.4809	1.0000	0.2835	0.7866	0.7514	0.1236	0.1745	0.4704	0.2696	0.1953
14	0.4691	1.0000	0.2490	0.4608	0.6311	1.0000	0.3064	0.4959	0.5566	1.0000	0.3010	0.7627	0.7251	0.1382	0.1867	0.4651	0.3030	0.1837
15	0.4691	1.0000	0.2490	0.4608	0.6311	1.0000	0.3064	0.4959	0.5566	1.0000	0.3010	0.7627	0.7251	0.1382	0.1867	0.4651	0.3030	0.1837
16	0.4389	1.0000	0.2103	0.4082	0.5822	0.8750	0.2612	0.4896	0.4809	1.0000	0.2835	0.7866	0.7514	0.1236	0.1745	0.4704	0.2696	0.1953
17	0.4679	1.0000	0.2779	0.5022	0.5857	1.0000	0.3580	0.4143	0.6282	0.6610	0.2727	0.5214	0.4294	0.1360	0.1604	0.2953	0.3059	0.1020
18	0.4389	1.0000	0.2103	0.4082	0.5822	0.8750	0.2612	0.4896	0.4809	1.0000	0.2835	0.7866	0.7514	0.1236	0.1745	0.4704	0.2696	0.1953
Mean	0.3807	0.9208	0.2084	0.3895	0.5587	0.8873	0.2577	0.4028	0.5186	0.8735	0.2429	0.5777	0.5519	0.1091	0.1405	0.3354	0.2339	0.1345
Ranking	10	1	15	9	5	2	12	8	7	3	13	4	6	18	16	11	14	17

S1 Table 2. The Cross Efficiency Matrix Derived from the CRS Benevolent Model

DMU	1	2	3	4	5	6	7	8	9	10	11	12	13	14	15	16	17	18
1	0.4691	1.0000	0.2490	0.4608	0.6311	1.0000	0.3064	0.4959	0.5566	1.0000	0.3010	0.7627	0.7251	0.1382	0.1867	0.4651	0.3030	0.1837
2	0.4679	1.0000	0.2779	0.5022	0.5857	1.0000	0.3580	0.4143	0.6282	0.6610	0.2727	0.5214	0.4294	0.1360	0.1604	0.2953	0.3059	0.1020
3	0.4679	1.0000	0.2779	0.5022	0.5857	1.0000	0.3580	0.4143	0.6282	0.6610	0.2727	0.5214	0.4294	0.1360	0.1604	0.2953	0.3059	0.1020
4	0.4679	1.0000	0.2779	0.5022	0.5857	1.0000	0.3580	0.4143	0.6282	0.6610	0.2727	0.5214	0.4294	0.1360	0.1604	0.2953	0.3059	0.1020
5	0.4691	1.0000	0.2490	0.4608	0.6311	1.0000	0.3064	0.4959	0.5566	1.0000	0.3010	0.7627	0.7251	0.1382	0.1867	0.4651	0.3030	0.1837
6	0.4679	1.0000	0.2779	0.5022	0.5857	1.0000	0.3580	0.4143	0.6282	0.6610	0.2727	0.5214	0.4294	0.1360	0.1604	0.2953	0.3059	0.1020
7	0.4679	1.0000	0.2779	0.5022	0.5857	1.0000	0.3580	0.4143	0.6282	0.6610	0.2727	0.5214	0.4294	0.1360	0.1604	0.2953	0.3059	0.1020
8	0.4691	1.0000	0.2490	0.4608	0.6311	1.0000	0.3064	0.4959	0.5566	1.0000	0.3010	0.7627	0.7251	0.1382	0.1867	0.4651	0.3030	0.1837
9	0.1827	1.0000	0.1330	0.2671	0.6291	1.0000	0.1459	0.2647	0.6577	1.0000	0.1421	0.2233	0.2963	0.0413	0.0289	0.0696	0.0395	0.0443
10	0.4691	1.0000	0.2490	0.4608	0.6311	1.0000	0.3064	0.4959	0.5566	1.0000	0.3010	0.7627	0.7251	0.1382	0.1867	0.4651	0.3030	0.1837
11	0.4691	1.0000	0.2490	0.4608	0.6311	1.0000	0.3064	0.4959	0.5566	1.0000	0.3010	0.7627	0.7251	0.1382	0.1867	0.4651	0.3030	0.1837
12	0.4389	1.0000	0.2103	0.4082	0.5822	0.8750	0.2612	0.4896	0.4809	1.0000	0.2835	0.7866	0.7514	0.1236	0.1745	0.4704	0.2696	0.1953
13	0.4389	1.0000	0.2103	0.4082	0.5822	0.8750	0.2612	0.4896	0.4809	1.0000	0.2835	0.7866	0.7514	0.1236	0.1745	0.4704	0.2696	0.1953
14	0.4691	1.0000	0.2490	0.4608	0.6311	1.0000	0.3064	0.4959	0.5566	1.0000	0.3010	0.7627	0.7251	0.1382	0.1867	0.4651	0.3030	0.1837
15	0.4691	1.0000	0.2490	0.4608	0.6311	1.0000	0.3064	0.4959	0.5566	1.0000	0.3010	0.7627	0.7251	0.1382	0.1867	0.4651	0.3030	0.1837
16	0.4389	1.0000	0.2103	0.4082	0.5822	0.8750	0.2612	0.4896	0.4809	1.0000	0.2835	0.7866	0.7514	0.1236	0.1745	0.4704	0.2696	0.1953
17	0.4679	1.0000	0.2779	0.5022	0.5857	1.0000	0.3580	0.4143	0.6282	0.6610	0.2727	0.5214	0.4294	0.1360	0.1604	0.2953	0.3059	0.1020
18	0.4389	1.0000	0.2103	0.4082	0.5822	0.8750	0.2612	0.4896	0.4809	1.0000	0.2835	0.7866	0.7514	0.1236	0.1745	0.4704	0.2696	0.1953
Mean	0.4461	1.0000	0.2436	0.4522	0.6050	0.9722	0.3047	0.4545	0.5693	0.8870	0.2788	0.6576	0.6086	0.1288	0.1665	0.3877	0.2819	0.1513
Ranking	10	1	15	9	6	2	12	8	7	3	14	4	5	18	16	11	13	17

S1 Table 3. The Cross Efficiency Matrix Derived from the VRS Input Aggressive Model

DMU	1	2	3	4	5	6	7	8	9	10	11	12	13	14	15	16	17	18
1	1.0000	1.0000	0.7576	0.9029	0.8609	0.4613	1.0000	0.4640	0.9129	0.7769	1.0000	0.7937	0.1521	0.3411	0.2027	0.6881	0.2787	0.4520
2	0.6636	1.0000	0.2567	0.4330	0.1839	0.0375	1.0000	0.1692	0.3310	0.0656	0.5418	0.2304	0.0185	0.0987	0.0300	0.0813	0.0628	0.1231
3	0.8725	0.3075	0.8656	0.9244	1.0000	0.7085	1.0000	0.4546	0.9884	1.0000	0.9054	0.8109	0.1976	0.4476	0.3159	0.9427	0.4798	0.4265
4	1.0000	0.7860	0.8288	0.9421	1.0000	0.6659	1.0000	0.5225	1.0000	0.9893	0.9311	0.9096	0.2049	0.4278	0.3073	0.9336	0.4688	0.4564
5	0.5304	0.3769	0.4598	0.6451	1.0000	0.6040	0.4564	0.4120	1.0000	1.0000	0.6193	0.6577	0.1753	0.1632	0.1116	0.4002	0.1278	0.3209
6	0.2403	0.3385	0.2245	0.3955	0.8895	1.0000	0.2296	0.2757	1.0000	1.0000	0.2186	0.2379	0.1633	0.0664	0.0442	0.1057	0.0597	0.0560
7	0.1563	0.0205	0.3646	0.1717	0.0992	0.0575	1.0000	0.0292	0.1560	0.0522	0.2508	0.0369	0.0069	0.1132	0.0284	0.0635	0.0530	0.0245
8	0.4367	1.0000	0.2185	0.4199	0.5892	1.0000	0.2718	0.5148	0.5055	1.0000	0.2761	0.7574	1.0000	0.1239	0.1745	0.4425	0.2795	0.1772
9	0.6097	0.1496	0.8075	0.8082	0.6717	0.4173	1.0000	0.1985	1.0000	0.3825	0.7091	0.2348	0.0509	0.2396	0.1100	0.2551	0.1715	0.1149
10	0.0259	0.2607	0.0178	0.0353	0.1645	0.3335	0.0159	0.0730	0.1082	1.0000	0.0270	0.0778	1.0000	0.0065	0.0056	0.0213	0.0057	0.0200
11	0.6664	0.2154	0.7957	0.6533	0.6326	0.3244	1.0000	0.2693	0.6587	0.5648	1.0000	0.5005	0.0985	0.2985	0.1442	0.5241	0.1833	0.3731
12	1.0000	1.0000	0.7203	0.9130	0.9665	0.6259	0.8874	0.5541	0.9308	1.0000	0.8873	0.9202	0.2174	0.3885	0.2957	0.9305	0.4417	0.4740
13	0.0000	0.0000	0.0000	0.0000	0.0000	0.0000	0.0000	0.0000	0.0000	0.0000	0.0000	0.0000	1.0000	0.0000	0.0000	0.0000	0.0000	0.0000
14	0.8403	0.2995	0.8525	0.9142	1.0000	0.7469	0.9493	0.4512	1.0000	1.0000	0.8410	0.7864	0.2001	0.2497	0.3144	0.9041	0.4863	0.3902
15	0.4367	1.0000	0.2185	0.4199	0.5892	1.0000	0.2718	0.4884	0.5055	1.0000	0.2761	0.7574	1.0000	0.1239	0.2040	0.4425	0.2795	0.1772
16	0.8597	0.3169	0.8522	0.8455	0.9463	0.6131	0.9459	0.4534	0.8646	1.0000	0.9597	0.8616	0.1990	0.4469	0.3049	1.0000	0.4357	0.4970
17	0.4011	1.0000	0.2147	0.4174	0.4954	1.0000	0.2858	0.3915	0.5224	0.6091	0.2224	0.4926	1.0000	0.1067	0.1334	0.2437	0.3095	0.0888
18	0.4199	1.0000	0.1964	0.3896	0.5607	0.9134	0.2460	0.4848	0.4611	1.0000	0.2668	0.7732	1.0000	0.1157	0.1675	0.4472	0.2597	0.1967
Mean	0.5644	0.5595	0.4807	0.5684	0.6472	0.5838	0.6422	0.3448	0.6636	0.7467	0.5518	0.5466	0.4269	0.2088	0.1608	0.4681	0.2435	0.2427
Ranking	7	8	11	6	3	5	4	14	2	1	9	10	13	17	18	12	15	16

S1 Table 4. The Cross Efficiency Matrix Derived from the VRS Input Benevolent Model

DMU	1	2	3	4	5	6	7	8	9	10	11	12	13	14	15	16	17	18
1	1.0000	0.7860	0.8288	0.9658	1.0000	0.6659	1.0000	0.5225	1.0000	0.9893	0.9311	0.9096	0.2049	0.4278	0.3073	0.9336	0.4688	0.4564
2	1.0000	1.0000	0.7436	0.9534	0.9972	0.6880	0.9137	0.5566	1.0000	1.0000	0.8570	0.9443	0.2178	0.3893	0.3018	0.8947	0.4675	0.4328
3	0.8725	0.3075	0.8656	0.9244	1.0000	0.7085	1.0000	0.4546	0.9884	1.0000	0.9054	0.8109	0.1976	0.4476	0.3159	0.9427	0.4798	0.4265
4	1.0000	0.7860	0.8288	0.9421	1.0000	0.6659	1.0000	0.5225	1.0000	0.9893	0.9311	0.9096	0.2049	0.4278	0.3073	0.9336	0.4688	0.4564
5	1.0000	0.7860	0.8288	0.9658	1.0000	0.6659	1.0000	0.5225	1.0000	0.9893	0.9311	0.9096	0.2049	0.4278	0.3073	0.9336	0.4688	0.4564
6	0.7814	1.0000	0.5664	0.8432	0.9567	1.0000	0.6798	0.5462	1.0000	1.0000	0.5583	0.7985	0.2719	0.2827	0.2800	0.6497	0.4835	0.2439
7	1.0000	0.7860	0.8288	0.9658	1.0000	0.6659	1.0000	0.5225	1.0000	0.9893	0.9311	0.9096	0.2049	0.4278	0.3073	0.9336	0.4688	0.4564
8	0.4367	1.0000	0.2185	0.4199	0.5892	1.0000	0.2718	0.5148	0.5055	1.0000	0.2761	0.7574	1.0000	0.1239	0.1745	0.4425	0.2795	0.1772
9	1.0000	0.7860	0.8288	0.9658	1.0000	0.6659	1.0000	0.5225	1.0000	0.9893	0.9311	0.9096	0.2049	0.4278	0.3073	0.9336	0.4688	0.4564
10	1.0000	0.7640	0.8327	0.9588	1.0000	0.6577	1.0000	0.5228	0.9870	1.0000	0.9470	0.9211	0.2068	0.4325	0.3089	0.9533	0.4661	0.4711
11	1.0000	0.7344	0.8360	0.9356	0.9868	0.6128	1.0000	0.5165	0.9484	1.0000	1.0000	0.9338	0.2036	0.4311	0.2947	0.9608	0.4240	0.5086
12	1.0000	1.0000	0.7203	0.9130	0.9665	0.6259	0.8874	0.5541	0.9308	1.0000	0.8873	0.9202	0.2174	0.3885	0.2957	0.9305	0.4417	0.4740
13	0.4367	1.0000	0.2185	0.4199	0.5892	1.0000	0.2718	0.4884	0.5055	1.0000	0.2761	0.7574	1.0000	0.1239	0.1745	0.4425	0.2795	0.1772
14	0.8403	0.2995	0.8525	0.9142	1.0000	0.7469	0.9493	0.4512	1.0000	1.0000	0.8410	0.7864	0.2001	0.2497	0.3144	0.9041	0.4863	0.3902
15	0.4367	1.0000	0.2185	0.4199	0.5892	1.0000	0.2718	0.4884	0.5055	1.0000	0.2761	0.7574	1.0000	0.1239	0.2040	0.4425	0.2795	0.1772
16	0.9473	0.5351	0.8608	0.9019	0.9693	0.6133	1.0000	0.4876	0.9109	1.0000	0.9955	0.9036	0.2014	0.4519	0.3070	1.0000	0.4448	0.5084
17	0.4011	1.0000	0.2147	0.4174	0.4954	1.0000	0.2858	0.3915	0.5224	0.6091	0.2224	0.4926	1.0000	0.1067	0.1334	0.2437	0.3095	0.0888
18	0.4199	1.0000	0.1964	0.3896	0.5607	0.9134	0.2460	0.4848	0.4611	1.0000	0.2668	0.7732	1.0000	0.1157	0.1675	0.4472	0.2597	0.1967
Mean	0.8096	0.8095	0.6383	0.7898	0.8722	0.7720	0.7654	0.5039	0.8481	0.9753	0.7203	0.8392	0.4301	0.3226	0.2672	0.7735	0.4136	0.3641
Ranking	5	6	12	7	2	9	10	13	3	1	11	4	14	17	18	8	15	16

S1 Table 5. The Cross Efficiency Matrix Derived from the VRS Output Aggressive Model

DMU	1	2	3	4	5	6	7	8	9	10	11	12	13	14	15	16	17	18
1	1.0000	1.0000	0.7576	0.9029	0.8609	0.4613	1.0000	0.4640	0.9129	0.7769	1.0000	0.7937	0.1521	0.3411	0.2027	0.6881	0.2787	0.4520
2	0.0156	1.0000	0.0029	0.0151	0.0354	0.0312	0.0063	0.0341	0.0393	0.1015	0.0079	0.0258	1.0000	0.0011	0.0007	0.0020	0.0011	0.0043
3	0.8838	0.3271	0.8962	0.9087	1.0000	0.6379	1.0000	0.4561	0.9676	1.0000	1.0000	0.8352	0.1917	0.4228	0.2743	0.8995	0.3784	0.4814
4	1.0000	0.7860	0.8288	0.9658	1.0000	0.6659	1.0000	0.5225	1.0000	0.9893	0.9311	0.9096	0.2049	0.4278	0.3073	0.9336	0.4688	0.4564
5	0.5304	0.3769	0.4598	0.6451	1.0000	0.6040	0.4564	0.4120	1.0000	1.0000	0.6193	0.6577	0.1753	0.1632	0.1116	0.4002	0.1278	0.3209
6	0.0888	0.1959	0.0804	0.1479	0.4107	1.0000	0.0813	0.1415	0.4009	1.0000	0.0771	0.1095	1.0000	0.0245	0.0175	0.0414	0.0234	0.0211
7	0.1563	0.0205	0.3646	0.1717	0.0992	0.0575	1.0000	0.0292	0.1560	0.0522	0.2508	0.0369	0.0069	0.1132	0.0284	0.0635	0.0530	0.0245
8	1.0000	1.0000	0.7436	0.9534	0.9972	0.6880	0.9137	0.5566	1.0000	1.0000	0.8570	0.9443	0.2178	0.3893	0.3018	0.8947	0.4675	0.4328
9	0.2472	1.0000	0.2001	0.3819	0.8817	0.9408	0.2133	0.3057	1.0000	1.0000	0.2105	0.2613	0.1659	0.0590	0.0389	0.0943	0.0527	0.0593
10	0.0436	0.2641	0.0390	0.0749	0.2571	0.6235	0.0392	0.0791	0.2474	1.0000	0.0388	0.0552	1.0000	0.0114	0.0078	0.0188	0.0103	0.0106
11	0.6664	0.2154	0.7957	0.6533	0.6326	0.3244	1.0000	0.2693	0.6587	0.5648	1.0000	0.5005	0.0985	0.2985	0.1442	0.5241	0.1833	0.3731
12	1.0000	1.0000	0.7203	0.9130	0.9665	0.6259	0.8874	0.5541	0.9308	1.0000	0.8873	0.9739	0.2174	0.3885	0.2957	0.9305	0.4417	0.4740
13	2.21E-11	2.51E-10	1.51E-11	3.01E-11	1.43E-10	3.14E-10	1.35E-11	6.35E-11	9.31E-11	1.03E-09	2.29E-11	6.69E-11	1.00E+00	5.52E-12	4.73E-12	1.81E-11	4.80E-12	1.70E-11
14	0.8789	0.3151	0.8887	0.8595	0.9424	0.5978	1.0000	0.4476	0.8699	0.9801	0.9990	0.8513	0.1927	0.4620	0.3033	1.0000	0.4335	0.5046
15	0.8725	0.3075	0.8916	0.9244	1.0000	0.7085	1.0000	0.4546	0.9884	1.0000	0.9054	0.8109	0.1976	0.4476	0.3159	0.9427	0.4798	0.4265
16	0.8597	0.3169	0.8522	0.8455	0.9463	0.6131	0.9459	0.4534	0.8646	1.0000	0.9597	0.8616	0.1990	0.4469	0.3049	1.0000	0.4357	0.4970
17	0.6728	0.2530	0.6663	0.8231	0.9667	1.0000	0.7253	0.4263	1.0000	1.0000	0.5761	0.6469	0.2274	0.3228	0.2973	0.6993	0.5006	0.2311
18	0.9281	0.4661	0.8709	0.8921	0.9667	0.6119	1.0000	0.4773	0.9035	1.0000	1.0000	0.8911	0.1997	0.4541	0.3054	1.0000	0.4389	0.5093
Mean	0.6025	0.4914	0.5588	0.6155	0.7202	0.5662	0.6816	0.3380	0.7189	0.8036	0.6289	0.5648	0.3582	0.2652	0.1810	0.5629	0.2653	0.2933
Ranking	7	12	11	6	2	8	4	14	3	1	5	9	13	17	18	10	16	15

Note: Under the VRS assumption, the output-oriented efficiency measures obtained from the deaR package are equal to the reciprocals of the values presented in S1 Table 5.

S1 Table 6. The Cross Efficiency Matrix Derived from the VRS Output Benevolent Model

DMU	1	2	3	4	5	6	7	8	9	10	11	12	13	14	15	16	17	18
1	1.0000	0.7640	0.8327	0.9588	1.0000	0.6577	1.0000	0.5228	0.9870	1.0000	0.9470	0.9211	0.2068	0.4325	0.3089	0.9533	0.4661	0.4711
2	1.0000	1.0000	0.7737	0.9637	0.9121	0.6069	1.0000	0.4913	1.0000	0.8129	0.8434	0.7931	0.1685	0.3851	0.2685	0.7545	0.4366	0.3676
3	0.8838	0.3271	0.8962	0.9087	1.0000	0.6379	1.0000	0.4561	0.9676	1.0000	1.0000	0.8352	0.1917	0.4228	0.2743	0.8995	0.3784	0.4814
4	1.0000	0.7860	0.8288	0.9658	1.0000	0.6659	1.0000	0.5225	1.0000	0.9893	0.9311	0.9096	0.2049	0.4278	0.3073	0.9336	0.4688	0.4564
5	1.0000	0.7640	0.8327	0.9588	1.0000	0.6577	1.0000	0.5228	0.9870	1.0000	0.9470	0.9211	0.2068	0.4325	0.3089	0.9533	0.4661	0.4711
6	0.6728	0.2530	0.6663	0.8231	0.9667	1.0000	0.7253	0.4263	1.0000	1.0000	0.5761	0.6469	0.2274	0.3228	0.2973	0.6993	0.5006	0.2311
7	1.0000	0.7860	0.8288	0.9658	1.0000	0.6659	1.0000	0.5225	1.0000	0.9893	0.9311	0.9096	0.2049	0.4278	0.3073	0.9336	0.4688	0.4564
8	1.0000	1.0000	0.7436	0.9534	0.9972	0.6880	0.9137	0.5566	1.0000	1.0000	0.8570	0.9443	0.2178	0.3893	0.3018	0.8947	0.4675	0.4328
9	1.0000	0.7860	0.8288	0.9658	1.0000	0.6659	1.0000	0.5225	1.0000	0.9893	0.9311	0.9096	0.2049	0.4278	0.3073	0.9336	0.4688	0.4564
10	1.0000	0.7305	0.8344	0.9312	0.9799	0.6156	1.0000	0.5168	0.9372	1.0000	0.9890	0.9374	0.2054	0.4408	0.3059	0.9865	0.4486	0.5068
11	1.0000	0.7344	0.8360	0.9356	0.9868	0.6128	1.0000	0.5165	0.9484	1.0000	1.0000	0.9338	0.2036	0.4311	0.2947	0.9608	0.4240	0.5086
12	1.0000	1.0000	0.7203	0.9130	0.9665	0.6259	0.8874	0.5541	0.9308	1.0000	0.8873	0.9739	0.2174	0.3885	0.2957	0.9305	0.4417	0.4740
13	0.4199	1.0000	0.1964	0.3896	0.5607	0.9134	0.2460	0.4848	0.4611	1.0000	0.2668	0.7732	1.0000	0.1157	0.1675	0.4472	0.2597	0.1848
14	0.8789	0.3151	0.8887	0.8595	0.9424	0.5978	1.0000	0.4476	0.8699	0.9801	0.9990	0.8513	0.1927	0.4620	0.3033	1.0000	0.4335	0.5046
15	0.8725	0.3075	0.8916	0.9244	1.0000	0.7085	1.0000	0.4546	0.9884	1.0000	0.9054	0.8109	0.1976	0.4476	0.3159	0.9427	0.4798	0.4265
16	0.9473	0.5351	0.8608	0.9019	0.9693	0.6133	1.0000	0.4876	0.9109	1.0000	0.9955	0.9036	0.2014	0.4519	0.3070	1.0000	0.4448	0.5084
17	0.6728	0.2530	0.6663	0.8231	0.9667	1.0000	0.7253	0.4263	1.0000	1.0000	0.5761	0.6469	0.2274	0.3228	0.2973	0.6993	0.5006	0.2311
18	0.9281	0.4661	0.8709	0.8921	0.9667	0.6119	1.0000	0.4773	0.9035	1.0000	1.0000	0.8911	0.1997	0.4541	0.3054	1.0000	0.4389	0.5093
Mean	0.9042	0.6560	0.7776	0.8908	0.9564	0.6970	0.9165	0.4949	0.9384	0.9867	0.8657	0.8618	0.2488	0.3991	0.2930	0.8846	0.4441	0.4266
Ranking	5	12	10	6	2	11	4	13	3	1	8	9	18	16	17	7	14	15

Note: Under the VRS assumption, the output-oriented efficiency measures obtained from the deaR package are equal to the reciprocals of the values presented in S1 Table 6.
