# Supplementary material for: Decomposing DEA self-efficiency: A cross-efficiency-based quantity–price framework
Source: PLoS One. 2026 Aug 10;21(8):e0355649. doi: 10.1371/journal.pone.0355649 (PMC13456291; doi:10.1371/journal.pone.0355649)
Supplement: Supplementary file 2 [file pone.0355649.s002.pdf]

Supporting information

S2 Table. The Quantity Matrix

S2 Table 1. The Quantity Matrix Derived from the CRS Aggressive Cross Efficiency Matrix

DMU	1	2	3	4	5	6	7	8	9	10	11	12	13	14	15	16	17	18
1	1.0000	0.1226	1.7809	0.9738	0.7433	0.2540	1.4144	0.9458	0.4839	0.1654	1.5585	0.5858	0.6147	3.3942	2.5123	0.9702	1.5386	2.3962
2	8.1584	1.0000	24.5633	8.0643	5.0782	2.7390	14.5720	5.8672	4.5651	2.0072	14.2326	5.1336	6.1916	57.3864	61.0897	22.6785	37.9683	24.3873
3	0.5615	0.0407	1.0000	0.5534	0.4327	0.1869	0.7762	0.5803	0.2991	0.1306	0.9182	0.3775	0.4256	1.9189	1.5201	0.6487	0.9084	1.7131
4	1.0269	0.1240	1.8071	1.0000	0.7913	0.3385	1.4027	1.0614	0.5698	0.2443	1.6793	0.7070	0.7970	3.5095	2.7800	1.2149	1.6415	3.2083
5	1.3454	0.1969	2.3112	1.2637	1.0000	0.6096	1.8355	1.2725	1.0414	0.4487	2.0968	0.7826	0.8212	4.5665	3.3800	1.2960	1.9967	3.2010
6	3.9369	0.3651	5.3497	2.9538	1.6405	1.0000	4.6778	3.2174	1.5862	0.6616	5.2375	2.6604	2.1256	13.7982	14.2295	5.4368	9.5972	11.7501
7	0.7070	0.0686	1.2883	0.7129	0.5448	0.2138	1.0000	0.7307	0.3556	0.1403	1.1562	0.4775	0.5384	2.4162	1.9140	0.8206	1.1703	2.1670
8	1.0573	0.1704	1.7231	0.9422	0.7859	0.3108	1.3685	1.0000	0.5989	0.2641	1.6478	0.6362	0.6676	3.5887	2.6562	1.0535	1.4886	2.6021
9	2.0665	0.2191	3.3435	1.7551	0.9602	0.6305	2.8120	1.6697	1.0000	0.3830	2.9252	1.3418	1.1920	8.0034	8.2325	3.1077	5.8457	6.0500
10	6.0456	0.4982	7.6545	4.0929	2.2287	1.5115	7.1293	3.7871	2.6109	1.0000	7.3631	2.8065	2.2153	22.1064	20.6863	6.6653	12.9998	10.7324
11	0.6416	0.0703	1.0890	0.5955	0.4769	0.1909	0.8649	0.6069	0.3419	0.1358	1.0000	0.3772	0.3958	2.1778	1.6120	0.6246	0.9408	1.5426
12	1.7070	0.1948	2.6491	1.4145	1.2778	0.3759	2.0941	1.5719	0.7453	0.3563	2.6515	1.0000	1.0468	5.9267	4.2913	1.6723	2.2301	4.0285
13	1.6267	0.1615	2.3497	1.2547	1.2177	0.4704	1.8575	1.4980	0.8389	0.4514	2.5268	0.9553	1.0000	5.6480	4.0895	1.5976	1.9781	3.8485
14	0.2946	0.0174	0.5211	0.2849	0.2190	0.0725	0.4139	0.2787	0.1249	0.0452	0.4592	0.1687	0.1771	1.0000	0.7402	0.2794	0.4502	0.6901
15	0.3980	0.0164	0.6579	0.3597	0.2959	0.0703	0.5225	0.3765	0.1215	0.0483	0.6204	0.2330	0.2445	1.3510	1.0000	0.3859	0.5683	0.9531
16	1.0308	0.0441	1.5415	0.8231	0.7716	0.1839	1.2186	0.9492	0.3218	0.1500	1.6011	0.5980	0.6260	3.5789	2.5913	1.0000	1.2977	2.4089
17	0.6499	0.0263	1.1009	0.6092	0.5008	0.1042	0.8545	0.6718	0.1711	0.0769	1.0629	0.4484	0.5055	2.2212	1.7595	0.7706	1.0000	2.0349
18	0.4173	0.0410	0.5837	0.3117	0.3124	0.0851	0.4615	0.3843	0.1653	0.0932	0.6482	0.2482	0.2598	1.4490	1.0492	0.4151	0.4914	1.0000

S2 Table 2. The Quantity Matrix Derived from the CRS Benevolent Cross Efficiency Matrix

DMU	1	2	3	4	5	6	7	8	9	10	11	12	13	14	15	16	17	18
1	1.0000	0.4685	1.7809	0.9738	0.7433	0.4685	1.4144	0.9458	0.4839	0.4691	1.5585	0.5858	0.6147	3.3942	2.5123	0.9702	1.5386	2.3962
2	2.1345	1.0000	3.5983	1.9912	1.6448	1.0000	2.7930	2.2062	1.5557	1.2300	3.4907	1.5615	1.7604	7.2949	5.7786	2.6833	3.2686	7.0860
3	0.5615	0.2779	1.0000	0.5534	0.4327	0.2779	0.7762	0.5803	0.2991	0.3236	0.9182	0.3775	0.4256	1.9189	1.5201	0.6487	0.9084	1.7131
4	1.0269	0.5022	1.8071	1.0000	0.7913	0.5022	1.4027	1.0614	0.5698	0.5917	1.6793	0.7070	0.7970	3.5095	2.7800	1.2149	1.6415	3.2083
5	1.3454	0.6080	2.3112	1.2637	1.0000	0.6080	1.8355	1.2725	1.0414	0.6311	2.0968	0.7826	0.8212	4.5665	3.3800	1.2960	1.9967	3.2010
6	2.1345	1.0000	3.5983	1.9912	1.6448	1.0000	2.7930	2.2062	1.5557	1.2300	3.4907	1.4607	1.6467	7.2949	5.7786	2.5101	3.2686	6.6285
7	0.7070	0.3580	1.2883	0.7129	0.5448	0.3580	1.0000	0.7307	0.3556	0.4074	1.1562	0.4775	0.5384	2.4162	1.9140	0.8206	1.1703	2.1670
8	1.0573	0.4533	1.7231	0.9422	0.7859	0.4533	1.3685	1.0000	0.5989	0.4959	1.6478	0.6362	0.6676	3.5887	2.6562	1.0535	1.4886	2.6021
9	2.0665	0.6428	3.3435	1.7551	0.9602	0.6428	2.8120	1.6697	1.0000	0.6050	2.9252	1.3418	1.1920	8.0034	8.2325	3.1077	5.8457	6.0500
10	2.1319	0.8130	3.0906	1.6899	1.5846	0.8130	2.4546	2.0164	1.6529	1.0000	3.3226	1.2911	1.3548	7.2360	5.3559	2.1380	2.6700	5.2807
11	0.6416	0.2865	1.0890	0.5955	0.4769	0.2865	0.8649	0.6069	0.3419	0.3010	1.0000	0.3772	0.3958	2.1778	1.6120	0.6246	0.9408	1.5426
12	1.7070	0.6404	2.6491	1.4145	1.2778	0.6846	2.0941	1.5719	0.7453	0.7746	2.6515	1.0000	1.0468	5.9267	4.2913	1.6723	2.2301	4.0285
13	1.6267	0.5681	2.3497	1.2547	1.2177	0.6073	1.8575	1.4980	0.8389	0.7381	2.5268	0.9553	1.0000	5.6480	4.0895	1.5976	1.9781	3.8485
14	0.2946	0.1371	0.5211	0.2849	0.2190	0.1371	0.4139	0.2787	0.1249	0.1382	0.4592	0.1687	0.1771	1.0000	0.7402	0.2794	0.4502	0.6901
15	0.3980	0.1731	0.6579	0.3597	0.2959	0.1731	0.5225	0.3765	0.1215	0.1867	0.6204	0.2330	0.2445	1.3510	1.0000	0.3859	0.5683	0.9531
16	1.0308	0.3727	1.5415	0.8231	0.7716	0.3984	1.2186	0.9492	0.3218	0.4677	1.6011	0.5980	0.6260	3.5789	2.5913	1.0000	1.2977	2.4089
17	0.6499	0.3059	1.1009	0.6092	0.5008	0.3059	0.8545	0.6718	0.1711	0.3745	1.0629	0.4484	0.5055	2.2212	1.7595	0.7706	1.0000	2.0349
18	0.4173	0.1411	0.5837	0.3117	0.3124	0.1509	0.4615	0.3843	0.1653	0.1894	0.6482	0.2482	0.2598	1.4490	1.0492	0.4151	0.4914	1.0000

S2 Table 3. The Quantity Matrix Derived from the VRS Input Aggressive Cross Efficiency Matrix

DMU	1	2	3	4	5	6	7	8	9	10	11	12	13	14	15	16	17	18
1	1.0000	0.8146	1.1535	1.0842	0.7849	0.7218	0.3954	1.3521	0.8173	0.1828	0.8163	1.1701	0.0081	3.1411	3.2499	1.1177	2.1561	2.1730
2	1.2276	1.0000	1.1763	1.3881	1.4315	3.0064	0.1433	3.3883	0.6724	1.9936	0.6305	2.1719	0.0232	3.4862	12.7741	1.9746	7.1701	6.4265
3	0.8669	0.8501	1.0000	0.9076	0.6309	0.5238	0.5618	0.8990	0.8409	0.1243	0.8722	0.9141	0.0066	2.5698	1.7134	0.8846	1.1187	1.4235
4	0.9223	0.7204	1.1018	1.0000	0.7796	0.7481	0.4022	1.2127	0.8726	0.1834	0.8130	1.0137	0.0068	2.8398	2.5124	0.9237	1.6462	2.0218
5	1.2740	0.6986	1.5851	1.2827	1.0000	1.2135	0.4663	1.6665	0.8196	0.4056	1.0107	1.2636	0.0076	4.9540	5.0873	1.5377	3.5385	2.9803
6	1.3855	0.3326	1.9092	1.3368	0.8241	1.0000	0.5004	2.6542	0.6460	0.5775	1.2182	1.6908	0.0078	6.7118	10.5337	2.4088	7.3591	9.1023
7	2.5291	6.9771	1.7799	2.4864	2.1447	1.9985	1.0000	4.2506	2.5315	0.5519	1.9968	5.1091	0.0382	5.7962	6.8532	3.8591	4.1744	7.1405
8	0.7396	0.2951	1.1123	0.8246	0.6000	0.3768	0.2353	1.0000	0.4497	0.1939	0.7086	0.6398	0.0023	2.7402	2.6581	0.7263	1.5263	2.6759
9	1.2236	1.4872	1.1891	1.1460	1.2201	1.5479	0.3950	2.2238	1.0000	0.5319	0.9639	2.0755	0.0140	4.0882	4.7467	1.8412	3.1373	4.5156
10	5.4717	0.5016	8.0466	5.4517	2.4652	1.7316	1.8120	5.1580	1.8802	1.0000	4.5712	3.7366	0.0032	24.8124	29.6785	6.8494	18.6560	15.9463
11	1.2250	1.5861	1.1466	1.2300	0.9895	0.8209	0.5008	1.4111	1.0375	0.2188	1.0000	1.3881	0.0101	3.3590	3.0640	1.3532	1.9800	1.9066
12	0.8546	0.4604	1.0940	0.9865	0.7914	0.5914	0.1957	1.5631	0.4818	0.2676	0.7204	1.0000	0.0065	2.7313	3.3994	0.9230	1.8209	2.7622
13	123.3301	43.0115	151.0855	147.4900	132.4190	127.7718	26.2110	440.7256	71.3702	316.2278	99.2372	153.6975	1.0000	283.1183	700.2212	141.0702	568.3848	712.9357
14	0.3184	0.2868	0.3891	0.3521	0.2019	0.1490	0.1725	0.3649	0.2446	0.0403	0.2977	0.3661	0.0035	1.0000	0.6947	0.3513	0.4207	0.6135
15	0.3077	0.0783	0.5836	0.3980	0.1966	0.0949	0.1459	0.3762	0.2107	0.0337	0.3264	0.2942	0.0014	1.4395	1.0000	0.3749	0.5608	0.9899
16	0.8947	0.5064	1.1305	1.0826	0.6503	0.4151	0.2591	1.3768	0.5431	0.1460	0.7390	1.0834	0.0071	2.8466	2.6674	1.0000	1.3442	2.1386
17	0.4638	0.1395	0.8939	0.6075	0.2826	0.1359	0.2396	0.6552	0.3187	0.0536	0.5050	0.5492	0.0018	2.3771	1.7832	0.7440	1.0000	2.1454
18	0.4602	0.1556	0.7025	0.4946	0.3355	0.1099	0.1400	0.3737	0.2215	0.0627	0.5245	0.3620	0.0014	1.6300	1.0102	0.4676	0.4661	1.0000

S2 Table 4. The Quantity Matrix Derived from the VRS Input Benevolent Cross Efficiency Matrix

DMU	1	2	3	4	5	6	7	8	9	10	11	12	13	14	15	16	17	18
1	1.0000	1.1279	1.1028	1.0484	1.0000	1.0833	1.0000	1.2741	1.0000	1.0054	1.0363	1.0930	1.4597	2.8049	2.6395	1.0073	1.6626	2.1625
2	0.8866	1.0000	0.6912	0.9355	0.8879	1.2056	0.9275	1.8681	0.8866	0.8741	0.9257	1.0728	2.1427	1.7555	4.0309	0.7734	2.6288	3.4270
3	0.9068	1.4468	1.0000	0.9076	0.8470	0.8319	0.8470	0.8990	0.8519	0.8490	0.8940	0.9141	0.9784	2.5698	1.7134	0.8890	1.1187	1.4235
4	0.9539	1.0690	1.1018	1.0000	0.9539	1.0922	0.9539	1.2127	0.9539	0.9555	0.9730	1.0137	1.3894	2.8398	2.5124	0.9540	1.6462	2.0218
5	1.0000	1.1263	1.1806	1.0484	1.0000	1.1986	1.0000	1.4799	1.0000	1.0054	1.0295	1.0745	1.6955	3.0599	3.0659	1.0189	1.8477	2.4989
6	0.9231	0.8294	1.2021	0.9156	0.8343	1.0000	0.9897	1.8857	0.8160	0.8110	1.0476	0.9230	1.9177	3.2531	4.1843	0.9716	2.5849	4.3627
7	1.0000	1.0782	1.1806	1.0484	1.0000	1.0104	1.0000	1.0052	1.0000	1.0054	1.0363	1.0297	1.1516	2.9812	2.0824	1.0350	1.4034	1.6553
8	0.7849	0.5353	1.1123	0.8246	0.6757	0.5303	0.9949	1.0000	0.7295	0.5188	0.9814	0.6398	0.5014	2.7402	2.6581	0.7532	1.5263	2.6759
9	1.0000	1.1279	1.1738	1.0484	1.0000	1.2255	1.0000	1.3707	1.0000	0.9988	1.0093	1.0545	1.5704	3.0599	2.8398	0.9878	1.8974	2.2661
10	0.9947	1.1440	1.1779	1.0465	0.9947	1.2330	0.9947	1.9275	1.0012	1.0000	1.0276	1.0862	2.1992	3.0431	3.9842	1.0242	2.0548	3.2848
11	0.9649	1.0802	1.1185	1.0278	0.9714	0.9545	0.9649	1.0190	0.9908	0.9731	1.0000	1.0162	1.1645	2.7951	2.1435	1.0179	1.3018	1.6329
12	0.9149	0.9322	1.0940	0.9865	0.9306	1.0835	0.9712	1.5631	0.9483	0.9207	0.9841	1.0000	1.7906	2.7313	3.3994	0.9453	1.8209	2.7622
13	0.6851	0.4667	1.0221	0.7197	0.5898	0.5215	0.8684	1.9942	0.6368	0.4547	0.8588	0.5585	1.0000	2.5433	5.3009	0.6747	3.3996	5.3564
14	0.3565	0.5696	0.3891	0.3521	0.3268	0.3074	0.3354	0.3649	0.3268	0.3286	0.3578	0.3661	0.3932	1.0000	0.6947	0.3533	0.4207	0.6135
15	0.3789	0.2481	0.5836	0.3980	0.3262	0.2390	0.4802	0.3762	0.3521	0.2510	0.4665	0.2942	0.1886	1.4395	1.0000	0.3762	0.5608	0.9899
16	0.9927	1.2930	1.1248	1.0482	0.9814	1.0292	0.9662	1.3277	1.0124	0.9764	0.9824	1.0579	1.4822	2.8309	2.6584	1.0000	1.3304	2.1145
17	0.6015	0.3804	0.8939	0.6075	0.5412	0.3869	0.7126	0.6552	0.5270	0.4867	0.7682	0.5492	0.2942	2.3771	1.7832	0.7517	1.0000	2.1454
18	0.4624	0.2918	0.7025	0.4946	0.4002	0.2292	0.6041	0.3737	0.4413	0.3044	0.6124	0.3620	0.1867	1.6300	1.0102	0.4729	0.4661	1.0000

S2 Table 5. The Quantity Matrix Derived from the VRS Output Aggressive Cross Efficiency Matrix

DMU	1	2	3	4	5	6	7	8	9	10	11	12	13	14	15	16	17	18
1	1.0000	0.1251	1.1410	1.0709	0.7849	0.4388	0.3954	1.9678	0.5204	0.2368	0.8163	1.1374	0.0000	2.3615	3.6911	1.1177	2.1959	2.0080
2	7.9967	1.0000	11.2707	7.3344	3.2607	2.5044	1.8054	7.2638	5.0472	1.6132	5.2110	6.3103	0.0000	25.3906	37.2771	12.5221	21.4389	14.6682
3	0.8765	0.0887	1.0000	0.9200	0.6419	0.3360	0.5717	1.6203	0.4305	0.1870	0.8445	0.8909	0.0000	2.0193	3.0370	0.9215	1.7756	1.7842
4	0.9338	0.1363	1.0869	1.0000	0.7893	0.4632	0.4072	1.7793	0.6073	0.2703	0.8232	0.9976	0.0000	2.0494	3.0324	0.9352	1.8406	1.9253
5	1.2740	0.3067	1.5578	1.2669	1.0000	0.8246	0.4663	2.0852	0.9390	0.5071	1.0107	1.2283	0.0000	3.5354	5.3254	1.5377	3.8872	2.4323
6	2.2787	0.3993	2.9761	2.1589	1.2127	1.0000	0.8409	2.9556	1.5319	0.7896	2.0519	2.4228	0.0000	7.2608	11.3331	3.8463	9.2491	7.5496
7	2.5291	0.5539	1.7493	2.4558	2.1447	1.1892	1.0000	7.4955	1.1691	0.8668	1.9968	4.9662	0.0000	4.3734	10.5623	3.8591	5.2297	8.9477
8	0.5082	0.1377	0.6172	0.5620	0.4796	0.3383	0.1334	1.0000	0.4125	0.2098	0.4182	0.5791	0.0000	1.1769	1.6291	0.5311	1.0070	1.0978
9	1.9215	0.1981	2.3229	1.6465	1.0650	0.6528	0.8554	2.4242	1.0000	0.4974	1.7691	1.9125	0.0000	5.6505	8.9668	3.0272	6.1546	5.4718
10	4.2235	0.6199	5.3467	3.6993	1.9721	1.2665	1.1537	4.7666	2.0106	1.0000	3.8135	4.3130	0.0000	13.6585	20.1389	7.2859	13.9218	13.6330
11	1.2250	0.1919	1.1842	1.2148	0.9895	0.4873	0.5008	2.3910	0.5653	0.2622	1.0000	1.3492	0.0000	2.6914	4.4582	1.3532	2.5059	2.2942
12	0.8792	0.1585	1.1225	1.0024	0.8141	0.4127	0.2014	1.7269	0.5229	0.2319	0.7412	1.0000	0.0000	2.1492	2.9076	0.9496	1.6881	1.8962
13	8.30E+04	6.32E+04	1.19E+05	8.40E+04	3.50E+04	5.64E+04	2.26E+04	7.85E+04	4.22E+04	3.12E+04	6.55E+04	5.78E+04	1.0000	2.75E+05	3.64E+05	1.05E+05	3.08E+05	1.52E+05
14	0.4235	0.0394	0.4952	0.4879	0.2829	0.1377	0.2287	0.8497	0.1770	0.0732	0.3716	0.4653	0.0000	1.0000	1.4690	0.4544	0.8290	0.9035
15	0.2709	0.0268	0.3293	0.3298	0.1878	0.0882	0.0947	0.6138	0.1115	0.0497	0.2243	0.3439	0.0000	0.6807	1.0000	0.3197	0.6253	0.6664
16	0.8947	0.0799	1.0852	1.0693	0.6503	0.2600	0.2591	1.8829	0.3303	0.1373	0.7390	1.0531	0.0000	2.2009	3.1282	1.0000	1.7906	1.9877
17	0.4554	0.0466	0.5632	0.5433	0.2573	0.1081	0.1912	0.9931	0.1625	0.0718	0.3991	0.5924	0.0000	1.2062	1.5991	0.5585	1.0000	1.3662
18	0.4980	0.0682	0.5605	0.5194	0.4111	0.1325	0.1118	0.9109	0.1828	0.0734	0.4359	0.5274	0.0000	1.1068	1.5005	0.5031	0.7320	1.0000

S2 Table 6. The Quantity Matrix Derived from the VRS Output Benevolent Cross Efficiency Matrix

DMU	1	2	3	4	5	6	7	8	9	10	11	12	13	14	15	16	17	18
1	1.0000	1.1440	1.0883	1.0392	1.0000	1.0113	1.0000	1.8538	1.0066	1.0000	1.0276	1.0558	1.4251	2.0973	2.9903	0.9969	1.6981	1.9670
2	0.8741	1.0000	0.6868	0.9190	0.9153	0.6457	0.8866	1.9123	0.8866	0.9479	0.9332	1.1378	2.4362	1.3307	1.9038	0.8421	1.0759	1.5778
3	0.9189	1.4561	1.0000	0.9200	0.8639	0.9675	0.8618	1.6203	0.8762	0.8648	0.8656	0.8909	0.9584	2.0193	3.0370	0.9261	1.7756	1.7842
4	0.9623	1.0881	1.0869	1.0000	0.9623	1.0926	0.9658	1.7793	0.9658	0.9534	0.9851	0.9976	1.3549	2.0494	3.0324	0.9659	1.8406	1.9253
5	1.0000	1.0926	1.1576	1.0392	1.0000	1.2123	1.0000	1.8512	1.0066	0.9899	1.0208	1.0379	1.6468	2.1717	3.2013	1.0083	2.0356	2.0074
6	0.9888	1.5488	1.0336	0.9152	0.8248	1.0000	0.9582	1.7028	0.8160	0.7846	1.0314	0.9967	2.0043	2.0020	2.7465	0.9365	1.9977	2.2801
7	1.0000	1.1279	1.1603	1.0355	1.0000	1.0437	1.0000	1.7725	1.0000	1.0054	1.0363	1.0008	1.0957	2.2494	3.2094	1.0350	1.7581	2.0743
8	0.5394	0.5229	0.6172	0.5620	0.5402	0.5873	0.5642	1.0000	0.5393	0.5363	0.5792	0.5791	1.1130	1.1769	1.6291	0.5508	1.0070	1.0978
9	0.9935	1.1279	1.1413	1.0355	0.9935	1.2255	1.0000	1.8543	1.0000	0.9733	1.0093	1.0250	1.4999	2.0979	3.1908	0.9878	2.0644	1.9716
10	1.0000	1.0549	1.1564	1.0489	1.0102	1.2745	0.9947	1.8645	1.0274	1.0000	1.0055	1.0466	2.2063	2.1938	3.2170	1.0068	2.1101	1.9683
11	0.9731	1.0716	1.1553	1.0152	0.9796	0.9696	0.9649	1.7265	0.9908	0.9945	1.0000	0.9878	1.1447	2.2395	3.1188	1.0179	1.6476	1.9648
12	0.9472	0.8789	1.1225	1.0024	0.9635	1.0033	0.9992	1.7269	0.9756	0.9555	1.0124	1.0000	1.8612	2.1492	2.9076	0.9725	1.6881	1.8962
13	0.7017	0.4105	1.0434	0.7381	0.6072	0.4989	0.9127	0.8985	0.6667	0.4532	0.8736	0.5373	1.0000	1.8983	1.9326	0.6711	1.3225	1.4569
14	0.4768	0.7515	0.4952	0.4879	0.4605	0.4995	0.4446	0.8497	0.4767	0.4558	0.4465	0.4653	0.5268	1.0000	1.4690	0.4569	0.8290	0.9035
15	0.3344	0.5253	0.3293	0.3298	0.3124	0.3641	0.3116	0.6138	0.3134	0.3108	0.3206	0.3439	0.5174	0.6807	1.0000	0.3208	0.6253	0.6664
16	1.0032	1.1874	1.0798	1.0353	0.9917	1.0679	0.9662	1.8157	1.0124	0.9932	0.9824	1.0283	1.4901	2.1887	3.1176	1.0000	1.7722	1.9653
17	0.5889	0.9294	0.5632	0.5433	0.4913	0.5006	0.5688	0.9931	0.4844	0.4739	0.6070	0.5924	0.7561	1.2062	1.5991	0.5643	1.0000	1.3662
18	0.5084	0.6338	0.5605	0.5194	0.4982	0.4386	0.4821	0.9109	0.5072	0.5080	0.5089	0.5274	0.6864	1.1068	1.5005	0.5088	0.7320	1.0000
