# Supplementary material for: Decomposing DEA self-efficiency: A cross-efficiency-based quantity–price framework
Source: PLoS One. 2026 Aug 10;21(8):e0355649. doi: 10.1371/journal.pone.0355649 (PMC13456291; doi:10.1371/journal.pone.0355649)
Supplement: Supplementary file 3 [file pone.0355649.s003.pdf]

Supporting information

S3 Table. The Price Matrix

S3 Table 1. The Price Matrix Derived from the CRS Aggressive Cross Efficiency Matrix

DMU	1	2	3	4	5	6	7	8	9	10	11	12	13	14	15	16	17	18
1	1.0000	3.8268	0.9477	0.9591	1.0000	1.8467	0.9262	1.0000	1.4739	2.8358	1.0000	1.0179	1.0154	1.0000	1.0000	1.0279	0.9965	1.0025
2	0.2613	1.0000	0.1465	0.2469	0.3120	0.3651	0.1917	0.3437	0.3331	0.4982	0.2334	0.2476	0.2149	0.1261	0.0877	0.0937	0.0861	0.2100
3	1.0552	6.8264	1.0000	1.0000	1.0178	1.4867	1.0000	0.9656	1.4129	2.1273	1.0056	0.9359	0.8690	1.0480	0.9792	0.9108	1.0000	0.8308
4	1.0426	4.0501	1.0000	1.0000	1.0057	1.4835	1.0000	0.9541	1.3403	2.0555	0.9937	0.9031	0.8385	1.0355	0.9676	0.8789	1.0000	0.8017
5	1.0000	3.2047	0.9825	0.9943	1.0000	1.0353	0.9603	1.0000	0.9214	1.4065	1.0000	1.0252	1.0227	1.0000	1.0000	1.0352	1.0331	1.0097
6	0.5415	2.7390	0.6726	0.6741	0.9659	1.0000	0.5971	0.6267	0.9586	1.5115	0.6344	0.4778	0.6261	0.5244	0.3764	0.3910	0.3406	0.4359
7	1.0796	5.2173	1.0000	1.0000	1.0414	1.6748	1.0000	0.9880	1.5309	2.5525	1.0289	0.9532	0.8850	1.0723	1.0019	0.9276	1.0000	0.8462
8	1.0000	2.9098	1.0357	1.0481	1.0000	1.5957	1.0122	1.0000	1.2592	1.8782	1.0000	0.9911	0.9887	1.0000	1.0000	1.0008	1.0890	0.9761
9	0.6785	3.0023	0.7078	0.7461	1.0853	1.0432	0.6532	0.7942	1.0000	1.7171	0.7470	0.6231	0.7342	0.5946	0.4279	0.4499	0.3677	0.5567
10	0.3526	2.0072	0.4701	0.4865	0.7110	0.6616	0.3918	0.5324	0.5824	1.0000	0.4512	0.4530	0.6007	0.3273	0.2589	0.3190	0.2514	0.4772
11	1.0000	4.2836	0.9944	1.0064	1.0000	1.5763	0.9719	1.0000	1.3387	2.2161	1.0000	1.0145	1.0120	1.0000	1.0000	1.0245	1.0456	0.9992
12	0.9824	4.0381	1.0685	1.1073	0.9755	2.0927	1.0491	1.0090	1.6049	2.2076	0.9857	1.0000	1.0000	0.9604	0.9817	1.0000	1.1529	1.0000
13	0.9848	4.6527	1.1507	1.1925	0.9778	1.5973	1.1299	1.0115	1.3619	1.6647	0.9881	1.0000	1.0000	0.9627	0.9841	1.0000	1.2417	1.0000
14	1.0000	7.9306	0.9542	0.9657	1.0000	1.9069	0.9326	1.0000	1.6818	3.0550	1.0000	1.0413	1.0387	1.0000	1.0000	1.0515	1.0033	1.0255
15	1.0000	11.4061	1.0212	1.0335	1.0000	2.6568	0.9981	1.0000	2.3372	3.8624	1.0000	1.0186	1.0161	1.0000	1.0000	1.0286	1.0738	1.0032
16	0.9728	10.6673	1.0979	1.1378	0.9660	2.5573	1.0781	0.9992	2.2227	3.1351	0.9761	1.0000	1.0000	0.9510	0.9722	1.0000	1.1847	1.0000
17	1.0035	11.6162	1.0000	1.0000	0.9680	2.9362	1.0000	0.9183	2.7194	3.9772	0.9564	0.8674	0.8054	0.9967	0.9313	0.8441	1.0000	0.7700
18	0.9975	4.7618	1.2036	1.2473	0.9904	2.2943	1.1818	1.0245	1.7962	2.0956	1.0008	1.0000	1.0000	0.9751	0.9968	1.0000	1.2987	1.0000

S3 Table 2. The Price Matrix Derived from the CRS Benevolent Cross Efficiency Matrix

DMU	1	2	3	4	5	6	7	8	9	10	11	12	13	14	15	16	17	18
1	1.0000	1.0012	0.9477	0.9591	1.0000	1.0012	0.9262	1.0000	1.4739	1.0000	1.0000	1.0179	1.0154	1.0000	1.0000	1.0279	0.9965	1.0025
2	0.9988	1.0000	1.0000	1.0000	0.9634	1.0000	1.0000	0.9139	0.9774	0.8130	0.9518	0.8142	0.7560	0.9919	0.9268	0.7923	1.0000	0.7228
3	1.0552	1.0000	1.0000	1.0000	1.0178	1.0000	1.0000	0.9656	1.4129	0.8589	1.0056	0.9359	0.8690	1.0480	0.9792	0.9108	1.0000	0.8308
4	1.0426	1.0000	1.0000	1.0000	1.0057	1.0000	1.0000	0.9541	1.3403	0.8487	0.9937	0.9031	0.8385	1.0355	0.9676	0.8789	1.0000	0.8017
5	1.0000	1.0380	0.9825	0.9943	1.0000	1.0380	0.9603	1.0000	0.9214	1.0000	1.0000	1.0252	1.0227	1.0000	1.0000	1.0352	1.0331	1.0097
6	0.9988	1.0000	1.0000	1.0000	0.9634	1.0000	1.0000	0.9139	0.9774	0.8130	0.9518	0.8704	0.8081	0.9919	0.9268	0.8470	1.0000	0.7726
7	1.0796	1.0000	1.0000	1.0000	1.0414	1.0000	1.0000	0.9880	1.5309	0.8788	1.0289	0.9532	0.8850	1.0723	1.0019	0.9276	1.0000	0.8462
8	1.0000	1.0942	1.0357	1.0481	1.0000	1.0942	1.0122	1.0000	1.2592	1.0000	1.0000	0.9911	0.9887	1.0000	1.0000	1.0008	1.0890	0.9761
9	0.6785	1.0232	0.7078	0.7461	1.0853	1.0232	0.6532	0.7942	1.0000	1.0870	0.7470	0.6231	0.7342	0.5946	0.4279	0.4499	0.3677	0.5567
10	1.0000	1.2300	1.1643	1.1783	1.0000	1.2300	1.1379	1.0000	0.9199	1.0000	1.0000	0.9847	0.9823	1.0000	1.0000	0.9944	1.2242	0.9698
11	1.0000	1.0506	0.9944	1.0064	1.0000	1.0506	0.9719	1.0000	1.3387	1.0000	1.0000	1.0145	1.0120	1.0000	1.0000	1.0245	1.0456	0.9992
12	0.9824	1.2283	1.0685	1.1073	0.9755	1.1490	1.0491	1.0090	1.6049	1.0156	0.9857	1.0000	1.0000	0.9604	0.9817	1.0000	1.1529	1.0000
13	0.9848	1.3228	1.1507	1.1925	0.9778	1.2374	1.1299	1.0115	1.3619	1.0180	0.9881	1.0000	1.0000	0.9627	0.9841	1.0000	1.2417	1.0000
14	1.0000	1.0081	0.9542	0.9657	1.0000	1.0081	0.9326	1.0000	1.6818	1.0000	1.0000	1.0413	1.0387	1.0000	1.0000	1.0515	1.0033	1.0255
15	1.0000	1.0789	1.0212	1.0335	1.0000	1.0789	0.9981	1.0000	2.3372	1.0000	1.0000	1.0186	1.0161	1.0000	1.0000	1.0286	1.0738	1.0032
16	0.9728	1.2621	1.0979	1.1378	0.9660	1.1807	1.0781	0.9992	2.2227	1.0057	0.9761	1.0000	1.0000	0.9510	0.9722	1.0000	1.1847	1.0000
17	1.0035	1.0000	1.0000	1.0000	0.9680	1.0000	1.0000	0.9183	2.7194	0.8169	0.9564	0.8674	0.8054	0.9967	0.9313	0.8441	1.0000	0.7700
18	0.9975	1.3836	1.2036	1.2473	0.9904	1.2943	1.1818	1.0245	1.7962	1.0311	1.0008	1.0000	1.0000	0.9751	0.9968	1.0000	1.2987	1.0000

S3 Table 3. The Price Matrix Derived from the VRS Input Aggressive Cross Efficiency Matrix

DMU	1	2	3	4	5	6	7	8	9	10	11	12	13	14	15	16	17	18
1	1.0000	1.2276	1.0015	0.9789	1.2740	1.3855	2.5291	1.4366	1.2236	5.4717	1.2250	0.9288	123.3301	1.2751	1.5087	0.8947	1.4983	2.3391
2	0.8146	1.0000	0.9821	0.7646	0.6986	0.3326	6.9771	0.5733	1.4872	0.5016	1.5861	0.5003	43.0115	1.1489	0.3838	0.5064	0.4506	0.7909
3	0.9985	1.0182	1.0000	1.0123	1.3721	1.6526	1.5407	1.8702	1.0293	6.9652	0.9925	1.0291	130.7812	1.3491	2.4771	0.9786	2.4996	3.0907
4	1.0215	1.3078	0.9879	1.0000	1.2085	1.2594	2.3426	1.5090	1.0797	5.1363	1.1588	1.0100	138.9576	1.3288	1.8386	1.0199	1.8489	2.3685
5	0.7849	1.4315	0.7288	0.8275	1.0000	0.8241	2.1447	1.1655	1.2201	2.4652	0.9895	0.8600	132.4190	0.8085	0.9638	0.6503	0.9130	1.7055
6	0.7218	3.0064	0.6051	0.7940	1.2135	1.0000	1.9985	0.7318	1.5479	1.7316	0.8209	0.6427	127.7718	0.5967	0.4655	0.4151	0.4390	0.5584
7	0.3954	0.1433	0.6490	0.4269	0.4663	0.5004	1.0000	0.4570	0.3950	1.8120	0.5008	0.2127	26.2110	0.6910	0.7155	0.2591	0.7739	0.7118
8	0.6961	1.7444	0.5347	0.6627	0.8580	1.3665	2.1883	1.0000	1.1449	2.6555	0.7265	0.8745	226.8986	0.7525	0.9496	0.7088	1.0897	0.9779
9	0.8173	0.6724	0.9715	0.9262	0.8196	0.6460	2.5315	0.8735	1.0000	1.8802	1.0375	0.5236	71.3702	0.9797	1.0329	0.5431	1.0297	1.1256
10	0.1828	1.9936	0.1436	0.1947	0.4056	0.5775	0.5519	0.3766	0.5319	1.0000	0.2188	0.2908	316.2278	0.1614	0.1652	0.1460	0.1732	0.3187
11	0.8163	0.6305	1.0076	0.8630	1.0107	1.2182	1.9968	1.3765	0.9639	4.5712	1.0000	0.7829	99.2372	1.1924	1.6002	0.7390	1.6316	2.6659
12	1.0767	1.9986	0.9717	0.9901	1.1628	1.5559	4.7014	1.1435	1.9099	3.4384	1.2773	1.0000	141.4314	1.3494	1.3272	0.9969	1.6326	1.6933
13	0.0081	0.0232	0.0076	0.0072	0.0076	0.0078	0.0382	0.0044	0.0140	0.0032	0.0101	0.0071	1.0000	0.0141	0.0070	0.0071	0.0057	0.0071
14	0.7842	0.8704	0.7412	0.7526	1.2369	1.6758	1.4472	1.3289	1.0207	6.1950	0.8387	0.7411	70.6865	1.0000	1.7622	0.7107	1.9173	2.0685
15	0.6628	2.6053	0.4037	0.5439	1.0376	2.1484	1.3977	1.0530	0.9681	6.0530	0.6249	0.7534	142.8120	0.5675	1.0000	0.5440	1.1750	1.0472
16	1.1177	1.9746	1.0219	0.9804	1.5377	2.4088	3.8591	1.4108	1.8412	6.8494	1.3532	1.0031	141.0702	1.4070	1.8382	1.0000	2.4034	2.3767
17	0.6674	2.2194	0.4001	0.5409	1.0953	2.2779	1.2922	0.9177	0.9711	5.7748	0.6129	0.6125	175.9371	0.5216	0.8511	0.4161	1.0000	0.7333
18	0.4275	1.2644	0.3235	0.4222	0.5864	1.7908	1.4048	1.0226	0.8884	3.1373	0.3751	0.5906	140.2651	0.4834	0.9549	0.4207	1.3636	1.0000

S3 Table 4. The Price Matrix Derived from the VRS Input Benevolent Cross Efficiency Matrix

DMU	1	2	3	4	5	6	7	8	9	10	11	12	13	14	15	16	17	18
1	1.0000	0.8866	1.0475	1.0124	1.0000	0.9231	1.0000	1.5245	1.0000	0.9947	0.9649	0.9943	0.6851	1.4279	1.8576	0.9927	1.9431	2.3504
2	1.1279	1.0000	1.6714	1.1346	1.1263	0.8294	1.0782	1.0398	1.1279	1.1440	1.0802	1.0130	0.4667	2.2815	1.2164	1.2930	1.2289	1.4832
3	0.9546	0.5983	1.0000	1.0123	1.0220	1.0405	1.0220	1.8702	1.0160	1.0196	0.9682	1.0291	0.8847	1.3491	2.4771	0.9736	2.4996	3.0907
4	0.9877	0.8814	0.9879	1.0000	0.9877	0.8626	0.9877	1.5090	0.9877	0.9860	0.9683	1.0100	0.6781	1.3288	1.8386	0.9875	1.8489	2.3685
5	1.0000	0.8879	0.9785	1.0124	1.0000	0.8343	1.0000	1.3125	1.0000	0.9947	0.9714	1.0114	0.5898	1.3090	1.5992	0.9814	1.7485	2.0340
6	1.0833	1.2056	0.9611	1.1593	1.1986	1.0000	1.0104	1.0300	1.2255	1.2330	0.9545	1.1774	0.5215	1.2312	1.1718	1.0292	1.2498	1.1650
7	1.0000	0.9275	0.9785	1.0124	1.0000	0.9897	1.0000	1.9324	1.0000	0.9947	0.9649	1.0554	0.8684	1.3435	2.3546	0.9662	2.3020	3.0706
8	0.6559	0.9618	0.5347	0.6627	0.7619	0.9708	0.5175	1.0000	0.7057	0.9923	0.5246	0.8745	1.0267	0.7525	0.9496	0.6835	1.0897	0.9779
9	1.0000	0.8866	0.9842	1.0124	1.0000	0.8160	1.0000	1.4170	1.0000	1.0012	0.9908	1.0306	0.6368	1.3090	1.7266	1.0124	1.7027	2.2430
10	1.0054	0.8741	0.9808	1.0142	1.0054	0.8110	1.0054	1.0077	0.9988	1.0000	0.9731	1.0005	0.4547	1.3162	1.2306	0.9764	1.5722	1.5474
11	1.0363	0.9257	1.0328	1.0327	1.0295	1.0476	1.0363	1.9062	1.0093	1.0276	1.0000	1.0694	0.8588	1.4330	2.2874	0.9824	2.4816	3.1127
12	1.0058	0.9871	0.9717	0.9901	0.9888	0.8493	0.9475	1.1435	0.9703	0.9995	0.9351	1.0000	0.5139	1.3494	1.3272	0.9735	1.6326	1.6933
13	1.4597	2.1427	1.1303	1.4747	1.6955	1.9177	1.1516	0.9740	1.5704	2.1992	1.1645	1.9459	1.0000	1.5748	0.9250	1.4822	0.9503	0.9489
14	0.7003	0.4383	0.7412	0.7526	0.7640	0.8122	0.7443	1.3289	0.7640	0.7598	0.6979	0.7411	0.6350	1.0000	1.7622	0.7068	1.9173	2.0685
15	0.5383	0.8221	0.4037	0.5439	0.6253	0.8534	0.4247	1.0530	0.5792	0.8126	0.4372	0.7534	1.0811	0.5675	1.0000	0.5422	1.1750	1.0472
16	1.0073	0.7734	1.0271	1.0126	1.0189	0.9716	1.0350	1.4630	0.9878	1.0242	1.0179	1.0273	0.6747	1.4149	1.8444	1.0000	2.4284	2.4037
17	0.5146	0.8137	0.4001	0.5409	0.5719	0.8001	0.4344	0.9177	0.5873	0.6360	0.4030	0.6125	1.0523	0.5216	0.8511	0.4118	1.0000	0.7333
18	0.4255	0.6742	0.3235	0.4222	0.4916	0.8583	0.3257	1.0226	0.4458	0.6463	0.3213	0.5906	1.0538	0.4834	0.9549	0.4160	1.3636	1.0000

S3 Table 5. The Price Matrix Derived from the VRS Output Aggressive Cross Efficiency Matrix

DMU	1	2	3	4	5	6	7	8	9	10	11	12	13	14	15	16	17	18
1	1.0000	7.9967	0.9779	0.9669	1.2740	2.2787	2.5291	0.9131	1.9215	4.2235	1.2250	0.9028	83049.1338	0.9166	0.8576	0.8947	0.9097	0.9779
2	0.1251	1.0000	0.0990	0.1412	0.3067	0.3993	0.5539	0.2474	0.1981	0.6199	0.1919	0.1627	63169.2035	0.0852	0.0849	0.0799	0.0932	0.1339
3	1.0226	10.1010	1.0000	1.0087	1.3961	2.6673	1.5677	0.9938	2.0818	4.7919	1.0613	1.0329	106515.7555	0.9607	0.9341	0.9726	1.0083	0.9863
4	1.0342	7.0832	0.9914	1.0000	1.2235	2.0849	2.3717	0.9752	1.5901	3.5726	1.1732	0.9940	81128.3929	1.0200	1.0082	1.0326	1.0482	0.9850
5	0.7849	3.2607	0.7163	0.8173	1.0000	1.2127	2.1447	0.8616	1.0650	1.9721	0.9895	0.8359	35021.0539	0.6122	0.5944	0.6503	0.5139	0.8073
6	0.4388	2.5044	0.3749	0.4796	0.8246	1.0000	1.1892	0.6079	0.6528	1.2665	0.4873	0.4238	56417.9108	0.2981	0.2793	0.2600	0.2160	0.2601
7	0.3954	1.8054	0.6379	0.4216	0.4663	0.8409	1.0000	0.2397	0.8554	1.1537	0.5008	0.2068	22581.2018	0.4949	0.2997	0.2591	0.3820	0.2195
8	1.0952	4.0428	1.0062	1.0254	1.1606	1.6450	4.1718	1.0000	1.3493	2.6530	1.3308	0.9869	43692.0033	1.0236	1.0815	1.0480	1.1042	0.9955
9	0.5204	5.0472	0.4804	0.6289	0.9390	1.5319	1.1691	0.7412	1.0000	2.0106	0.5653	0.5369	42215.1663	0.3831	0.3530	0.3303	0.3246	0.3589
10	0.2368	1.6132	0.2087	0.2799	0.5071	0.7896	0.8668	0.3769	0.4974	1.0000	0.2622	0.2381	31228.7314	0.1585	0.1572	0.1373	0.1435	0.1440
11	0.8163	5.2110	0.9422	0.8523	1.0107	2.0519	1.9968	0.7515	1.7691	3.8135	1.0000	0.7610	65514.2604	0.8042	0.7100	0.7390	0.7972	0.8559
12	1.1077	6.1458	0.9681	1.0061	1.1963	2.3596	4.8367	1.0133	1.8626	4.2005	1.3141	1.0000	56245.5957	0.9809	1.0603	1.0256	1.1525	1.0086
13	0.0000	0.0000	0.0000	0.0000	0.0000	0.0000	0.0000	0.0000	0.0000	0.0000	0.0000	0.0000	1.0000	0.0000	0.0000	0.0000	0.0000	0.0000
14	1.0910	11.7304	1.0409	0.9804	1.6333	3.3545	2.0205	0.9770	2.6105	6.3102	1.2434	1.0195	126988.7582	1.0000	0.9955	1.0168	1.1133	1.0041
15	1.1660	11.7760	1.0705	0.9919	1.6823	3.5802	3.3367	0.9247	2.8326	6.3619	1.4084	0.9431	114868.2752	1.0045	1.0000	0.9882	1.0092	0.9308
16	1.1177	12.5221	1.0282	0.9684	1.5377	3.8463	3.8591	0.9542	3.0272	7.2859	1.3532	0.9750	104803.5884	0.9835	1.0119	1.0000	1.1157	0.9879
17	1.0992	10.7318	0.9918	0.9540	1.9458	4.6299	2.6179	0.9057	3.0808	6.9689	1.2544	0.8677	153968.6672	0.8983	0.9909	0.8963	1.0000	0.7195
18	1.0226	7.4699	1.0139	1.0152	1.2387	3.8447	4.5567	1.0045	2.7866	6.9427	1.1683	0.9915	77323.9649	0.9959	1.0743	1.0122	1.3899	1.0000

S3 Table 6. The Price Matrix Derived from the VRS Output Benevolent Cross Efficiency Matrix

DMU	1	2	3	4	5	6	7	8	9	10	11	12	13	14	15	16	17	18
1	1.0000	0.8741	1.0253	0.9964	1.0000	0.9888	1.0000	0.9692	0.9935	1.0000	0.9731	0.9725	0.7017	1.0321	1.0586	1.0032	1.1764	0.9983
2	1.1440	1.0000	1.6247	1.1267	1.0926	1.5488	1.1279	0.9395	1.1279	1.0549	1.0716	0.9024	0.4105	1.6265	1.6627	1.1874	1.8567	1.2445
3	0.9754	0.6155	1.0000	1.0087	1.0375	0.9263	1.0399	0.9938	1.0229	1.0364	1.0354	1.0329	0.9351	0.9607	0.9341	0.9677	1.0083	0.9863
4	1.0036	0.8875	0.9914	1.0000	1.0036	0.8839	1.0000	0.9752	1.0000	1.0129	0.9804	0.9940	0.7128	1.0200	1.0082	0.9998	1.0482	0.9850
5	1.0000	0.9153	0.9639	0.9964	1.0000	0.8248	1.0000	0.9706	0.9935	1.0102	0.9796	0.9892	0.6072	0.9967	0.9888	0.9917	0.9814	0.9782
6	1.0113	0.6457	1.0795	1.1314	1.2123	1.0000	1.0437	1.0552	1.2255	1.2745	0.9696	1.0301	0.4989	1.0812	1.1526	1.0679	1.0000	0.8612
7	1.0000	0.8866	0.9616	1.0000	1.0000	0.9582	1.0000	1.0137	1.0000	0.9947	0.9649	1.0259	0.9127	0.9623	0.9863	0.9662	1.1363	0.9467
8	1.0318	1.0643	1.0062	1.0254	1.0303	0.9477	0.9865	1.0000	1.0321	1.0378	0.9609	0.9869	0.5001	1.0236	1.0815	1.0106	1.1042	0.9955
9	1.0066	0.8866	0.9776	1.0000	1.0066	0.8160	1.0000	0.9689	1.0000	1.0274	0.9908	1.0017	0.6667	1.0317	0.9921	1.0124	0.9677	0.9960
10	1.0000	0.9479	0.9649	0.9872	0.9899	0.7846	1.0054	0.9636	0.9733	1.0000	0.9945	0.9811	0.4532	0.9866	0.9840	0.9932	0.9467	0.9976
11	1.0276	0.9332	0.9658	1.0200	1.0208	1.0314	1.0363	1.0406	1.0093	1.0055	1.0000	1.0395	0.8736	0.9665	1.0150	0.9824	1.2125	0.9994
12	1.0283	1.1082	0.9681	1.0061	1.0109	0.9708	0.9747	1.0133	0.9983	1.0193	0.9620	1.0000	0.5233	0.9809	1.0603	1.0015	1.1525	1.0086
13	1.4251	2.4362	1.0694	1.4029	1.6468	2.0043	1.0957	1.9997	1.4999	2.2063	1.1447	1.9110	1.0000	1.1402	1.6380	1.4901	1.5105	1.3478
14	0.9689	0.6148	1.0409	0.9804	1.0033	0.9249	1.0392	0.9770	0.9692	1.0135	1.0347	1.0195	0.8770	1.0000	0.9955	1.0112	1.1133	1.0041
15	0.9447	0.6014	1.0705	0.9919	1.0113	0.8676	1.0139	0.9247	1.0080	1.0163	0.9852	0.9431	0.6105	1.0045	1.0000	0.9849	1.0092	0.9308
16	0.9969	0.8421	1.0334	1.0002	1.0083	0.9365	1.0350	0.9896	0.9878	1.0068	1.0179	0.9985	0.6711	0.9890	1.0154	1.0000	1.1273	0.9991
17	0.8500	0.5386	0.9918	0.9540	1.0190	1.0000	0.8801	0.9057	1.0334	1.0563	0.8247	0.8677	0.6620	0.8983	0.9909	0.8871	1.0000	0.7195
18	1.0017	0.8035	1.0139	1.0152	1.0223	1.1611	1.0563	1.0045	1.0040	1.0024	1.0006	0.9915	0.7419	0.9959	1.0743	1.0009	1.3899	1.0000
