# Supplementary material for: Decomposing DEA self-efficiency: A cross-efficiency-based quantity–price framework
Source: PLoS One. 2026 Aug 10;21(8):e0355649. doi: 10.1371/journal.pone.0355649 (PMC13456291; doi:10.1371/journal.pone.0355649)
Supplement: Supplementary file 4 [file pone.0355649.s004.pdf]

Supporting information

S4. Aggregated indicators and corresponding rankings

S4 Table 1. Aggregated Indicators and Corresponding Rankings under Different Cross-Efficiency Measures

DMU	CRS Aggressive				CRS Benevolent				VRS Input Aggressive				VRS Input Benevolent				VRS Output Aggressive				VRS Output Benevolent			
	Mean	R	QI	R	Mean	R	QI	R	Mean	R	QI	R	Mean	R	QI	R	Mean	R	QI	R	Mean	R	QI	R
1	0.3807	10	0.0280	10	1.0000	1	0.1117	1	0.5644	7	0.0043	9	0.8096	5	0.0652	7	0.6025	7	9.4747E-06	9	0.9042	5	0.0669	6
2	0.9208	1	0.3335	1	0.9722	2	0.1099	2	0.5595	8	0.0098	4	0.8095	6	0.0683	4	0.4914	12	6.5961E-05	2	0.6560	12	0.0587	12
3	0.2084	15	0.0168	15	0.5693	7	0.0992	3	0.4807	11	0.0037	12	0.6383	12	0.0544	13	0.5588	11	8.1869E-06	12	0.7776	10	0.0613	11
4	0.3895	9	0.0318	8	0.8870	3	0.0980	4	0.5684	6	0.0040	10	0.7898	7	0.0625	9	0.6155	6	9.1811E-06	10	0.8908	6	0.0655	9
5	0.5587	5	0.0426	7	0.6576	4	0.0756	5	0.6472	3	0.0055	7	0.8722	2	0.0688	3	0.7202	2	1.4499E-05	7	0.9564	2	0.0693	2
6	0.8873	2	0.1078	3	0.6086	5	0.0711	6	0.5838	5	0.0072	5	0.7720	9	0.0709	2	0.5662	8	2.4315E-05	5	0.6970	11	0.0665	7
7	0.2577	12	0.0212	12	0.6050	6	0.0647	7	0.6422	4	0.0146	3	0.7654	10	0.0614	10	0.6816	4	2.8392E-05	4	0.9165	4	0.0670	5
8	0.4028	8	0.0312	9	0.4522	9	0.0527	8	0.3448	14	0.0029	14	0.5039	13	0.0490	14	0.3380	14	5.8514E-06	14	0.4949	13	0.0375	15
9	0.5186	7	0.0620	4	0.4545	8	0.0491	9	0.6636	2	0.0067	6	0.8481	3	0.0674	5	0.7189	3	1.8780E-05	6	0.9384	3	0.0686	3
10	0.8735	3	0.1458	2	0.4461	10	0.0472	10	0.7467	1	0.0190	2	0.9753	1	0.0743	1	0.8036	1	3.9850E-05	3	0.9867	1	0.0711	1
11	0.2429	13	0.0181	13	0.3877	11	0.0443	11	0.5518	9	0.0052	8	0.7203	11	0.0599	12	0.6289	5	1.1318E-05	8	0.8657	8	0.0653	10
12	0.5777	4	0.0459	5	0.3047	12	0.0364	12	0.5466	10	0.0037	11	0.8392	4	0.0661	6	0.5648	9	8.9157E-06	11	0.8618	9	0.0656	8
13	0.5519	6	0.0447	6	0.2819	13	0.0319	13	0.4269	13	0.9039	1	0.4301	14	0.0602	11	0.3582	13	9.9973E-01	1	0.2488	18	0.0450	13
14	0.1091	18	0.0078	18	0.2788	14	0.0300	14	0.2088	17	0.0013	17	0.3226	17	0.0214	18	0.2652	17	3.7807E-06	17	0.3991	16	0.0314	17
15	0.1405	16	0.0098	17	0.2436	15	0.0287	15	0.1608	18	0.0012	18	0.2672	18	0.0222	17	0.1810	18	2.4977E-06	18	0.2930	17	0.0224	18
16	0.3354	11	0.0249	11	0.1665	16	0.0179	16	0.4681	12	0.0035	13	0.7735	8	0.0645	8	0.5629	10	7.7868E-06	13	0.8846	7	0.0673	4
17	0.2339	14	0.0171	14	0.1513	17	0.0179	17	0.2435	15	0.0020	15	0.4136	15	0.0377	15	0.2653	16	4.0925E-06	16	0.4441	14	0.0376	14
18	0.1345	17	0.0109	16	0.1288	18	0.0137	18	0.2427	16	0.0015	16	0.3641	16	0.0256	16	0.2933	15	4.1784E-06	15	0.4266	15	0.0333	16

Note: Mean represents the arithmetic mean of each column in the cross-efficiency matrix, whereas R denotes the corresponding rank.
